# Transdiagnostic cognitive behavioral therapy for severe and persistent fatigue – a feasibility study in primary care

**DOI:** 10.1186/s12875-026-03346-x

**Published:** 2026-04-29

**Authors:** Frank Svärdman, Conrad Samuelsson, Ludwig Franke Föyen, Anna Oremark, Anna Högfeldt, Jacob Andersson Emad, Douglas Sjöwall, Christian Rück, Erik Hedman-Lagerlöf, Hans Knoop, Elin Lindsäter

**Affiliations:** 1 Division of Psychology, Department of Clinical Neuroscience, Karolinska Institutet, Stockholm, Sweden; 2 Academic Primary Care Center, Gustavsberg University Primary Care Center, Region Stockholm and Karolinska Institutet, Stockholm, Sweden; 3 Stress Research Institute, Department of Psychology, Stockholm University, Stockholm, Sweden; 4 Osher Center for Integrative Health, Department of Clinical Neuroscience, Karolinska Institutet, Stockholm, Sweden; 5 Region Stockholm, Stockholm, Sweden; 6 Family Medicine and Primary Care, Department of Neurobiology, Care Sciences and Society, Karolinska Institutet, Stockholm, Sweden; 7 Center for Neurodevelopmental Disorders at Karolinska Institutet (KIND), CAP Research Center, Region Stockholm, Stockholm, Sweden; 8 Centre for Psychiatry Research, Department of Clinical Neuroscience, Karolinska Institutet and Stockholm Healthcare Services, Stockholm, Sweden; 9 Department of Medical Psychology, Amsterdam University Medical Centre and Amsterdam Public Health Research Institute, University of Amsterdam, Amsterdam, The Netherlands

**Keywords:** Fatigue, Primary care, Feasibility study, Cognitive behavioral therapy (CBT), Transdiagnostic, Blended intervention

## Abstract

**Background:**

Fatigue is the main complaint of 5–10% of patients in primary care and is often associated with significant suffering and functional disability. Cognitive behavioral therapy (CBT) has demonstrated efficacy in reducing fatigue severity in diverse clinical populations in specialized healthcare settings, with common treatment mechanisms identified across conditions. This study is the first to evaluate the feasibility and acceptability of a transdiagnostic CBT (tCBT) for a heterogeneous sample of patients suffering from severe and persistent fatigue in a primary care context.

**Methods:**

A single-arm feasibility study was conducted in routine primary care. Adults with severe and persistent fatigue received a blended tCBT combining individual sessions and internet-based materials. Feasibility and acceptability were evaluated through adherence, completion, attrition, therapist fidelity, patient satisfaction, credibility, and negative effects. Preliminary effectiveness was evaluated by self-reported symptom change from pre- to post-treatment (6 months).

**Results:**

Eighteen participants were enrolled, of whom fourteen completed the full intervention. Treatment adherence and therapist fidelity were satisfactory, and data attrition was minimal. Participants reported high satisfaction and treatment credibility. No serious adverse events were reported. Half of the participants reported some negative effects of treatment, primarily consisting of transient increases in fatigue and sleep disturbances. Large within-group reductions were observed in self-reported fatigue severity and functional impairment over the 6-month treatment period.

**Conclusions:**

Preliminary findings suggest that tCBT for fatigue is feasible and acceptable in a primary care setting. Interpretation is limited by the small sample size and single-arm design. Larger randomized controlled trials are warranted to evaluate effectiveness and to determine which patients benefit most and through which mechanisms.

**Trial registration:**

Pre-registered on Clinicaltrials.gov (NCT06341751), registration date: 2024-03-13.

**Supplementary Information:**

The online version contains supplementary material available at 10.1186/s12875-026-03346-x.

## Introduction

Fatigue is a common and typically transient symptom in the general population, often arising in response to stress, illness, or lifestyle factors, and usually resolving with rest or recovery [1]. Approximately 20% of adults experience fatigue lasting several months [2], and 5–10% of primary care patients present fatigue as their main complaint [1, 3, 4]. A subset of individuals develops severe and persistent fatigue (lasting more than six months) that is not alleviated by rest, and substantial functional impairment [1, 2]. This clinical form of fatigue is linked to increased healthcare use and mortality [5] and represents the target population of the present study.

Although fatigue can arise from diverse medical and psychosocial conditions, it remains a subjective symptom that correlates poorly with objective disease markers [6, 7]. The broad differential etiology and absence of clear diagnostic tests, make assessment and management particularly challenging in primary care [8]. Clinicians often encounter patients with multifactorial or unclear causes of fatigue, for whom care is usually limited to medical rule-out, reassurance, and general lifestyle advice (e.g., sleep hygiene or stress reduction). Structured, evidence-based psychological interventions targeting fatigue are rarely available and many patients continue to experience persistent impairment [9, 10], highlighting the need for interventions that are both effective and feasible to deliver in routine practice.

Cognitive behavioral therapy (CBT) has shown promising effects in reducing fatigue severity across a range of medical conditions [11–14]. CBT in these studies is based on the premise that while an initial trigger, such as prolonged stress, an infection, or a chronic medical condition, may initiate fatigue, cognitive and behavioral processes play a key role in its maintenance [11–14]. Factors including fear-avoidance, excessive rest, catastrophizing, symptom focusing, and reduced self-efficacy have been identified as perpetuating fatigue and mediating treatment response across patient groups [13, 15]. The recurrence of these mechanisms across clinical populations supports the potential of a transdiagnostic treatment approach, in which shared maintaining processes are targeted irrespective of specific diagnoses or fatigue triggers. Such an approach could improve scalability and implementation by allowing a single intervention to address fatigue across diverse patient groups.

Importantly, most CBT trials have been conducted in specialized care settings and have focused on diagnosis-specific populations. Evidence on the feasibility of delivering CBT for fatigue in a broader primary care population remains limited, creating an implementation gap in routine care where structured psychological treatments are rarely available despite the high prevalence of fatigue.

This study aimed to investigate the feasibility, acceptability, and preliminary effectiveness of a transdiagnostic CBT (tCBT) for severe and persistent fatigue in primary care. To our knowledge, no prior studies have specifically evaluated a tCBT intervention targeting fatigue across mixed clinical presentations in this setting.

## Method

### Study design

This was a single-arm exploratory feasibility study designed to assess the acceptability and practicality of delivering a tCBT intervention in primary care. The study did not employ predefined progression thresholds for advancing to a randomized controlled trial (RCT); instead, feasibility outcomes were evaluated descriptively using indicators such as participant acceptability, therapist adherence, and practical aspects of treatment delivery. The study was conducted in accordance with the CONSORT guidelines for pilot and feasibility trials [16] (see Supplementary Material for CONSORT-checklist). It was approved by the Ethical Review Authority in Stockholm (Dnr 2024-00393-01) and was pre-registered on Clinicaltrials.gov (NCT06341751; registration date: 2024-03-13). Recruitment, treatment, and data collection were initiated in March 2024 and completed in January 2025.

### Recruitment procedure

The study was conducted at Gustavsberg Primary Care Center (PCC) in Stockholm, Sweden. Potential participants were referred to the study by general practitioners (GPs) at Gustavsberg PCC and three other PCCs in the same geographical area. Recruitment followed a pragmatic consecutive referral procedure. During the recruitment period, GPs at participating primary care centers identified potentially eligible patients during routine consultations and referred them to Gustavsberg PCC using routine referral procedures after an initial medical evaluation. A digital referral support system already used in routine care was available as a supplementary means to identify and refer potentially eligible patients. However, the system was not successfully implemented for study purposes and generated few referrals, contributing minimally to overall recruitment. No researcher-led screening or purposive selection was undertaken; all referred patients were assessed for eligibility. This approach was chosen to mirror routine clinical pathways and enhance ecological validity.

After a GP referral, potential participants were given information about the study on the study webpage, before providing digital informed consent. They subsequently completed an online screening consisting of sociodemographic and clinical background questions, as well as self-rated symptom- and function questionnaires.

After screening, eligible participants were scheduled for a comprehensive medical assessment with a designated GP at Gustavsberg PCC, followed by a structured psychiatric assessment with a licensed psychologist. The psychiatric assessment included the MINI 7.0 interview [17] to identify potential psychiatric disorders, and covered other domains of relevance for fatigue presentations, such as post-exertional malaise (PEM). Functional disability was assessed across multiple domains, including ability to work, home management, social and private leisure activities, and interpersonal relationships. After the clinical assessments, a decision was made regarding inclusion or exclusion to the study (see criteria below).

Included participants were assigned to a therapist and initiated the treatment shortly after inclusion. The therapists were employed at Gustavsbergs PCC, where all sessions were conducted. Collaboration with referring clinicians occurred through standard referral documentation and medical record systems rather than through formal co-located multidisciplinary teamwork. The treatment period was six months.

**Fig. 1:**
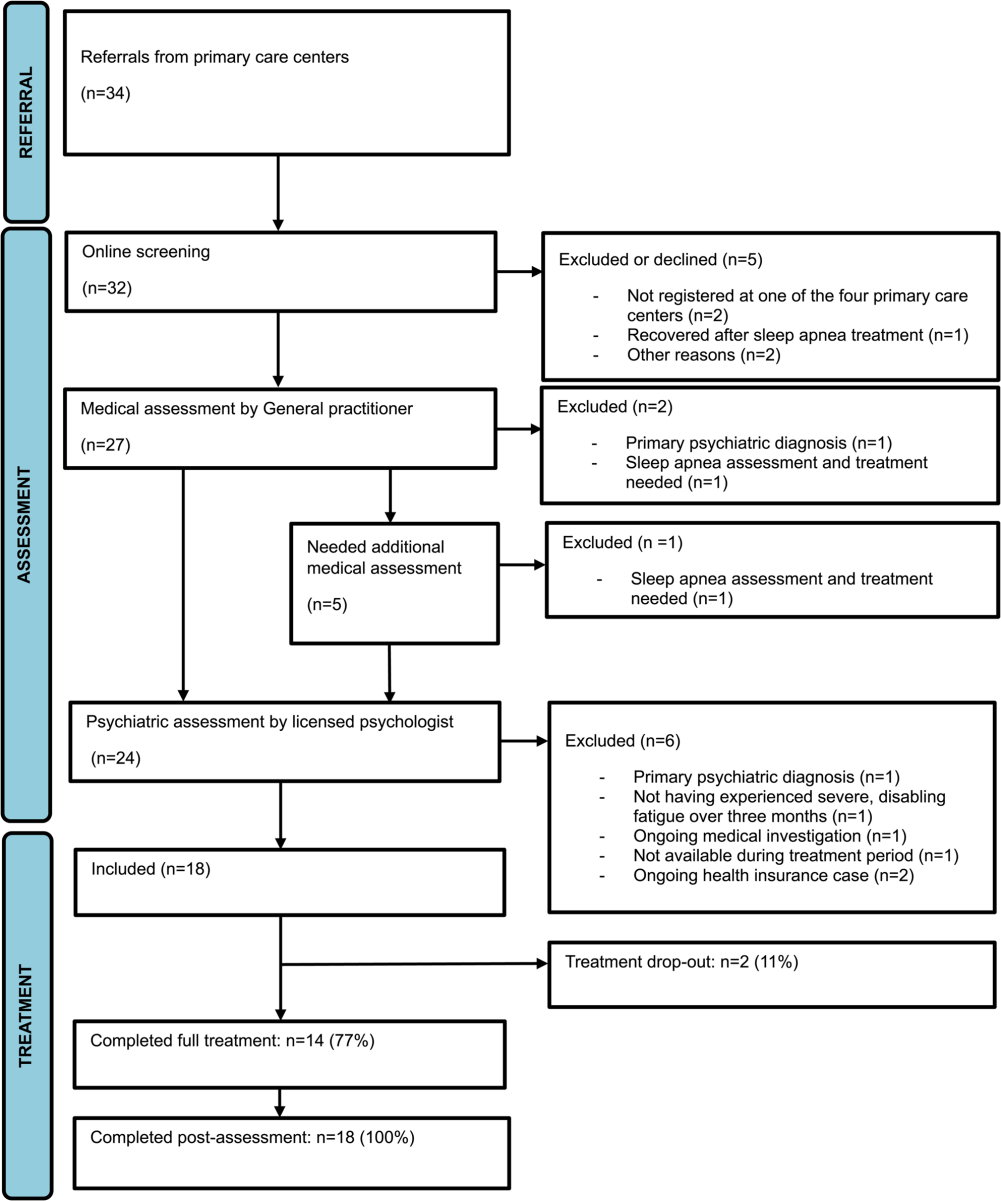
Flowchart of participant enrolment and retention

All data were collected via the digital research platform BASS4, a core facility at Karolinska Institutet, Sweden. A comprehensive overview of the recruitment procedure is presented in Fig. 1.

### Inclusion and exclusion criteria

Because fatigue in primary care spans multiple diagnoses and often lacks a single identifiable cause, eligibility was defined by symptom severity and functional impairment rather than diagnosis-specific criteria. In this study, a three-month symptom duration criterion was used to allow for inclusion of the broad range of fatigue presentations typical of a primary care population, and to enable earlier intervention.

Inclusion criteria were: (a) age 18–67 years, (b) severe and functionally disabling fatigue as a central symptom for at least 3 months, (c) that the fatigue was not a direct effect of an active disease process motivating another treatment (e.g., hypo-/hyperthyroidism, anemia, cancer, dementia) or the effect of medication, (d) regular access to a computer and to the internet, and (e) ability to read and write in Swedish.

Exclusion criteria were: (a) substance abuse disorder in the past 6 months, (b) current or past psychosis or bipolar disorder, (c) primary psychiatric disorder of such severity that it merits other treatments (e.g., obsessive compulsive disorder, moderate to severe depression, posttraumatic stress disorder), (d) elevated risk for suicide, (e) anorexia nervosa, (f) BMI > 40, (g) initiated or changed psychopharmacological medication (e.g., for depression or anxiety disorders) in the past month, (h) ongoing chemotherapy, (i) intellectual disability (or severe autism) that affects ability to work with the treatment, (j) self-harm, (k) pregnancy, (l) life circumstances that complicate or make treatment impossible (e.g., domestic violence or ongoing legal disputes), and (m) ongoing psychological treatment.

### Treatment description

The tCBT for fatigue was based on previous disorder-specific CBT protocols for fatigue that have shown efficacy in a range of clinical populations [12, 13, 18–20]. These CBT protocols are grounded in a cognitive behavioral framework and activity progression is introduced gradually and collaboratively, being neither strictly symptom-contingent or fixed. The main components of these CBT protocols were adapted in tCBT to suit different patient populations with fatigue. tCBT was also adapted to a blended format, meaning that individual face-to-face sessions were combined with an online treatment platform. The online treatment platform consisted of eight main modules and seven optional modules, including informative texts, illustrations, case examples, assignments, and worksheets. The intervention was designed to be flexible to suit the needs and preconditions of different participants. For that reason, there was no formal limit to the number of face-to-face sessions. However, approximately ten sessions were recommended. In addition, follow-ups and continuous support were provided in a text-message system integrated in the online treatment platform.

A detailed description of tCBT for fatigue according to the TIDieR guidelines [21] is provided in the supplementary material, Figure S1 and Table S6. In brief, the main perpetuating factors of fatigue that the treatment addresses include regulating a disrupted sleep-wake cycle, changing unhelpful beliefs about fatigue and excessive focusing on fatigue, and regulating uneven activity levels. The treatment is divided into three phases. The first phase focuses on presenting the CBT model of fatigue, setting goals, and addressing individual perpetuating factors. A session with a partner, next of kin or close friend, is included in this phase, given the role of interpersonal factors in maintaining fatigue within the CBT model. The second phase focuses on gradually increasing individual capacity in terms of physical, mental and social activity. Physical activity is introduced from each participant’s individual baseline and is gradually increased in small increments (e.g., approximately one minute of walking per day). Activity targets are individualized and continuously discussed with the patient throughout treatment. The third phase focuses on goal realization.

The internet platform was developed in BASS4 and could be accessed via computer, tablet, or smartphone. During the development of tCBT, a patient representative and two specialist licensed psychologists provided external feedback on the internet-delivered materials.

### Therapists and supervisors

The therapists were three licensed and one resident psychologist employed at Gustavsberg PCC. All received training and biweekly supervision by an experienced clinical psychologist (HK) who has been involved in the development of several disorder-specific CBT protocols targeting fatigue.

### Assessments and measurements

#### Feasibility and acceptability outcomes

Feasibility was assessed by investigating the assessment procedure (i.e. number of referrals, participant registrations, and included/excluded participants), number of face-to-face sessions, number of internet modules used, treatment adherence (rated by therapists and participants post-treatment), and data attrition. Feasibility was also assessed by analyzing therapist fidelity to the treatment. In total, 90·5% of all face-to-face therapy sessions were audio-recorded. A random sample of these were analyzed by author AO. A 7-item scoring instrument developed by the research team was used, where fidelity to central treatment components was rated on a 5-point Likert scale (“Not adherent” to “Fully adherent”). Average fidelity of three or higher was predefined as satisfactory. A detailed description of the fidelity analysis process and the fidelity rating instrument is available in the supplement.

Treatment completion was predefined as completing five or more face-to-face sessions and activation of the online module “Realizing goals” (see supplementary material for a detailed description of the treatment). Treatment drop-out was predefined as a participant informing the therapist of their decision to discontinue, which could occur at any point during the treatment period.

Treatment credibility was assessed three weeks after treatment start using the *Credibility/Expectancy Questionnaire (C-Scale*) [22]. Higher scores indicate higher treatment credibility (scale range: 0–50). Patient satisfaction was assessed post-treatment using the *Client Satisfaction Questionnaire (CSQ-8)* [23], where higher scores indicate greater treatment satisfaction (scale range: 8–32). Working alliance was assessed 15 weeks after treatment start using the *Working Alliance Inventory (WAI - short version)* [24], higher scores indicating a better working alliance with the therapist (scale range: 6–42). Negative effects of treatment were assessed post-treatment using *The Negative Effects of Treatment Questionnaire (NEQ-20)* [25]. The NEQ-20 addresses a wide range of negative effects, their severity (“*Not at all*” to “*Extremely*”), and attribution (treatment or other).

Feasibility and acceptability were additionally probed using a questionnaire developed by the research team with open-ended questions to therapists regarding their experience of working with the treatment, and one open-ended question to participants asking for general feedback regarding the treatment. These were completed after the treatment phase and are available in the supplementary material.

#### Preliminary effectiveness outcomes

Preliminary effectiveness was primarily assessed by investigating change in self-rated fatigue severity and functional impairment.

Fatigue severity was assessed pre, during (every third week) and post-treatment using the *Checklist Individual Strength (CIS) - Fatigue severity subscale (CIS-F)* [26]. CIS-F contains 8 items scored on a 7-point Likert scale (range 8–56; higher score indicating more severe fatigue). A score of 35 points on the CIS-F has, in previous studies, been suggested as a cut-off for severe fatigue [27]. Additionally, participants rated their improvement at the end of treatment using the following options: “*Completely recovered*”, “*Somewhat improved*”, “*No change*”, and “*Worse than before treatment*”. Functional impairment was assessed pre- and post-treatment using the *Work and Social Adjustment Scale (WSAS).* WSAS contains 5 items scored on an 8-point Likert scale (range 0–40; higher score indicating more severe disability) [28].

Additional exploratory effectiveness outcomes were administered pre, during (every third week) and post-treatment to evaluate the practicality and acceptability of administering the planned measurement battery, specifically completion rates and data quality. These findings will inform measurement planning for future randomized trials. All measures are presented in the supplementary material.

#### Target mechanisms of the treatment

Changes in cognitive and behavioral responses to fatigue have been hypothesized as central mechanisms of treatment effect. These were assessed pre, during (every third week) and post-treatment using the *Cognitive and Behavioral Responses to Symptoms Questionnaire (CBRQ)* 18-items [29]. The scale assesses six domains: Fear avoidance, Damage beliefs, Embarrassment avoidance, Symptom focusing, All-or-nothing behavior, and Resting behavior. Each item is rated on a 5-point Likert scale scored from 0 (strongly disagree) to 4 (strongly agree). Higher scores indicate more unhelpful cognitions and behaviors. Additional putative mechanisms were also assessed and are presented in the supplementary material.

### Data analysis

Feasibility and acceptability measurements are presented using descriptive statistics. All statistical analyses were performed using RStudio version 4.4.1 with packages ‘lme4’ version 1.1–37 and ‘lmerTest’ version 3.1-3. Preliminary effectiveness outcomes were analyzed using intention-to-treat linear mixed regression models, with significance level set at 0·05. Effect sizes were calculated as repeated measures Cohen’s *d* using the mean pre–post difference divided by the standard deviation of the difference scores. Calculations were performed using the “*repeated_measures_d”* function in the “*effectsize”* package (v 1.0.1.2.), specifying method “= “rm"” and 95% confidence intervals. Additionally, average point reduction and number of participants with a score below 35 points on the CIS-F were calculated.

Qualitative data from therapists were analyzed using conventional content analysis in accordance with Hsieh and Shannon [30]. Participants’ feedback on the treatment did not undergo analysis but is presented in full text in the supplementary material. All qualitative findings were used to complement and contextualize the feasibility results rather than to achieve analytic integration.

## Results

### Recruitment, assessment, and baseline characteristics of participants

Figure 1 displays detailed information about the participant flow throughout the study. In total, 34 referrals were received between March and May 2024. Of these, 27 individuals completed the study medical assessment, five of whom were referred back to their PCC for complementary medical assessments. Twenty-four individuals completed the psychiatric assessment, after which 18 were included.

Table 1 presents sociodemographic variables and baseline characteristics of included participants. Additional data collected at baseline is available in Supplementary Table S1. Of the 18 participants, eight reported a sudden onset of fatigue, and ten reported a gradual onset. The most common clinician-rated fatigue triggering event was an infection (*n* = 6), including covid-19, influenza and cold viruses. Other triggering events were stroke or head trauma (*n* = 2), stress (*n* = 4), and cancer/cancer treatment (*n* = 2). Four participants were categorized as “other triggering event”, including believed genetic disease, childhood trauma, combination of stress and infection, combination of infection and perimenopause. The level of activity disability was rated by clinicians as mild in five cases, moderate in ten, and severe in three (definitions of each level of activity disability is available in the supplementary material). Moreover, 13 (72%) of participants experienced PEM, which was clinically assessed during the psychiatric assessment (for a detailed description of PEM assessment procedures, see supplementary material).

**Table 1:** Participant characteristics at baseline (*N* = 18)

**Gender** ^**a**^	***n*** ** (%)**
Women	17 (94)
Men	1 (6)
**Age, mean (SD)**	44·83 (11·61)
**Highest Completed Education Level, n (%)**	
Elementary school	2 (11)
High school	4 (22)
Post-secondary education ≤ 3 years	2 (11)
Post-secondary education > 3 years	10 (56)
**Employment status, n (%)**	
Employed	10 (56)
Self-employed	2 (11)
Student	1 (6)
Unemployed	1 (6)
Other^b^	4 (22)
**Sick Leave, n (%)**	
Not on sick leave	6 (33)
Part-time sick leave	5 (28)
Full-time sick leave	7 (39)
**Duration of current sick leave period**	
Not receiving sickness benefit	9 (50)
< 6 months	3 (17)
6–12 months	2 (11)
> 12 months	4 (22)
**Duration of severe fatigue, years** **(median, IQR** ^**c**^ **, min – max)**	6 (IQR 2–4, min-max 1–26)

^a^ Non-binary and option to not answer was available but not registered by any participant

^b^Employment, other: work rehabilitation training, financial assistance

^c^IQR, Interquartile Range

### Treatment completion, drop-out rate, and data attrition

In total, 14 (78%) participants were categorized as full treatment completers. Of the four (22%) non-completers, two (11%) were categorized as dropouts. There was no data attrition pre- or post-treatment, and 97% of all process measurements during treatment were completed.

### Adherence to treatment and therapist fidelity

The mean treatment duration was 140 days (SD: 46) of the total 182 possible days (range 28–182). The average number of face-to-face sessions was 10·5 (SD: 2·7). The average therapist time spent on face-to-face sessions was 6·4 (SD: = 2) hours per participant. Most sessions occurred in the clinic, with 15% delivered via video. On average, participants engaged with 5·7 (SD: 4·2; median 6·5) of the eight mandatory internet modules, and nine participants (50%) used at least one optional module. A detailed description of activated modules is available in supplementary Table S7.

Participant adherence was generally higher in the beginning of treatment. Therapists reported high or complete adherence to the treatment for 12 (67%) participants in phase one, seven (39%) in phase two, and four (22%) in phase three. According to participants’ own ratings, 15 (83%) reported high or complete adherence in the first half of treatment, and eight (44%) in the second half.

Therapist fidelity was analyzed using 15 randomly selected recordings of treatment sessions. The overall fidelity was satisfactory (mean 3·6, SD 0·5, scale range 1–5), and 93% of sessions were rated as having satisfactory fidelity (≥ 3 or above on all domains). Fidelity was highest for adherence to the treatment content (mean: 4·29) whereas the lowest scores were observed for the component addressing unhelpful thoughts (mean: 2·9). An overview of the fidelity ratings is available in supplementary Table S9.

### Satisfaction, credibility, and negative effects

Participants generally reported high treatment satisfaction, as indicated by a mean CSQ-8 score of 26·1 (SD 5·9; scale range 8–32, where higher scores indicate higher satisfaction). Post-treatment, fifteen (83%) participants stated that they would recommend the treatment to a friend (CSQ-8, item four). Participants also reported high treatment credibility as assessed by the C-scale (mean: 36·5, SD 7·8; scale range 0–50, where higher scores indicate higher credibility). Working alliance with respective therapists was perceived as good, as indicated by a mean score on WAI of 36·1 (SD 7·64; scale range 6–42, where higher scores indicate better working alliance).

No serious adverse events were reported in the study. In total, nine participants (50%) reported some negative effects during the treatment that they attributed to the treatment. Increased fatigue (*n* = 4) and problems with sleep (*n* = 3) were the most common negative effects (effect severity: moderate or high). Complete results of the NEQ-20, including optional free text responses, are available in the supplementary material (Table S11).

### Participant and therapist feedback

Thirteen participants provided brief written comments post-treatment, which generally indicated positive experiences of the treatment structure and therapist support, alongside practical challenges related to time, energy demands, and sustaining engagement in later phases.

Therapist feedback was analyzed using content analysis. This analysis identified several themes related to feasibility and implementation, including perceived clinical usefulness of the intervention, factors influencing patient engagement, challenges associated with reduced adherence in later phases, and organizational or resource-related constraints. All participant comments as well as a detailed presentation of therapist themes are provided in the Supplementary Material.

### Preliminary effectiveness and putative mechanisms

Table 2 shows the results of self-rated fatigue (CIS-F), functional impairment (WSAS), and cognitive and behavioral responses to symptoms (CBRQ) pre- and post-treatment.

**Table 2:** Estimated means, standard deviations and effect sizes on preliminary effectiveness outcome measures from pre- to post-treatment

Measure	Pre	Post	*p*-value	Slope/b [95% CI]	Cohen’s d [95% CI]
	Mean (SD)	Mean (SD)			
Fatigue (CIS-F)	50·44 (5.49)	37·11 (11·80)	< 0·001	0·41 [0·29, 0·54]	1·35 [0·55, 2·16]
Functional impairment (WSAS)	28·50 (8·23)	18·44 (11·47)	< 0·001	0·42 [0·24, 0·60]	0·92 [0·46, 1·38]
All or nothing behavior (CBRQ)	8·56 (2·18)	6·00 (2·30)	< 0·001	0·09 [0·06, -0·13]	1·09 [0·26, 1·92]
Damage Beliefs (CBRQ)	7·39 (1·61)	4·39 (2·99)	< 0·001	0·09 [0·06, 0·12]	1·16 [0·45, 1·87]
Embarrassment Avoidance (CBRQ)	4·28 (2·78)	2·39 (3·22)	< 0·001	0·07 [0·03, 0·10]	0·60 [0·05, 1·15]
Fear Avoidance (CBRQ)	6·50 (3·19)	4·89 (3·77)	< 0·001	0·06 [0·03, 0·09]	0·44 [0·01, 0·86]
Resting Behavior (CBRQ)	7·89 (2·32)	2·33 (3·09)	< 0·001	0·15 [0·11, 0·19]	1·95 [0·70, 3·20]
Symptom Focusing (CBRQ)	8·00 (2·30)	4·67 (2·81)	< 0·001	0·14 [0·11, 0·17]	1·24 [0·41, 2·07]

*pre*, pre-treatment, *post* post-treatment, *CIS-F* Checklist Individual Strengths - Fatigue severity subscale, *WSAS* Work and Social Adjustment Scale, *CBRQ* Cognitive and Behavioral Responses to Symptoms Questionnaire

There was a large within-group reduction in self-rated fatigue from pre- to post-treatment (Cohen’s *d* 1·35). The average reduction pre- to post-treatment on the CIS-F was 13·34 points. Six (33%) participants scored below 35 on the CIS-F post-treatment, indicating that they were no longer severely fatigued. An additional two participants scored precisely 35 points on the CIS-F.

Fifteen participants (83%) reported being completely or somewhat improved at post-treatment (4 [22%] and 11 [61%] participants respectively). Two (11%) participants reported no change, and one (6%) reported feeling worse.

There was also a large within-group improvement in self-rated functional impairment from pre- to post-treatment (Cohen’s *d* 0·92). Regarding cognitive and behavioral responses to symptoms (CBRQ), large within-group reductions were found in All or nothing behavior, Damage Beliefs, Resting Behavior, and Symptom Focusing, while Embarrassment Avoidance and Fear Avoidance showed moderate within-group effect sizes.

The results of all additional exploratory self-rated symptom- and functioning outcomes, as well as ratings at all time points for process measures, are available in the supplementary material (Table S13, Table S14 and Figure S2).

## Discussion

This single-arm feasibility study suggests that the tCBT for fatigue, delivered in a blended format, can be implemented and is acceptable in routine primary care. Indicators of feasibility included minimal data attrition, low dropout rates, satisfactory therapist fidelity to the protocol, high credibility and satisfaction ratings, and the absence of serious adverse events. Therapists also reported that the blended and transdiagnostic format was workable within the primary care context. Treatment adherence was high during the initial phase but declined in later stages, highlighting the need for refinements to better sustain engagement throughout the intervention.

### Recruitment

Recruitment through routine primary care was feasible but was likely limited by existing workflows. Referrals depended on opportunistic GP identification and the digital support tool was rarely used, which might have reduced referral volume. As this exploratory feasibility study lacked predefined recruitment targets, these figures should be interpreted descriptively and not used to estimate recruitment rates for an RCT. Optimizing referral procedures and increasing assessment capacity are important considerations for future implementation and scalability.

### Acceptability and user experience

Participants rated the intervention as credible and satisfactory, and most participants would recommend the treatment to a friend with similar problems. A common limitation of satisfaction data is response bias from completers only [31]; notably, this study had no post-treatment data attrition, which strengthens confidence in acceptability signals. Additionally, written feedback from participants was generally positive. Of note, measures used in the study reflect global perceptions of the intervention and do not allow conclusions regarding the acceptability of specific components, the digital platform, or the blended delivery format.

Therapist reflections suggested that the transdiagnostic approach and the blended delivery format were workable within routine primary care. However, neither the digital platform nor the blended format was formally evaluated using component-specific acceptability or usability measures. Therefore, conclusions regarding these aspects should be considered exploratory. Therapists also noted a need for clearer guidance on integrating the digital platform with face-to-face sessions.

### Adherence and therapist fidelity

Treatment adherence was initially high but declined towards the later stages of the treatment. This pattern may reflect structural features of the blended format rather than reduced acceptability. Early phases included more frequent face-to-face contact and therapist guidance, whereas later phases relied more on independent digital work, which may have contributed to lower adherence. These results underscore the need to balance self-management with sufficient guidance. For a future RCT, strategies such as increased therapist check-ins, additional prompts, or enhanced digital support in later phases may help maintain engagement throughout the intervention.

Therapist fidelity was overall satisfactory. Addressing helpful and unhelpful thoughts was rated just below the cut-off for satisfaction, suggesting that therapists may need additional training to address this part of treatment.

### Safety and negative effects

No serious adverse events occurred. Half of the participants reported at least one negative effect during treatment, as assessed by the Negative Effects Questionnaire (NEQ). The most commonly reported effects were increases in fatigue and sleep disturbances, which is consistent with findings from previous CBT studies in fatigue and related conditions [12, 32–34]. The NEQ asks for adverse or unwanted experiences occurring at any point during treatment and does not distinguish between transient reactions and sustained deterioration. At post-treatment, however, 83% of participants reported being completely or somewhat improved, and only one participant reported overall deterioration. This pattern suggests that most reported negative effects were transient and occurred alongside perceived overall improvement.

### Preliminary effectiveness and putative mechanisms

Large within-group reductions were observed in fatigue and functional impairment, and in key cognitive–behavioral targets (all-or-nothing behavior, damage beliefs, resting behavior, and symptom focusing), mirroring patterns reported in trials of disorder-specific CBT for fatigue [13]. The proportion of participants with CIS-F scores < 35 at post-treatment was somewhat lower than in some prior trials [18, 20]. However, the Swedish CIS translation lacks population norms, complicating interpretation of the < 35 cut-off; this should be addressed in future work.

Findings of preliminary effectiveness should be interpreted cautiously given the small sample and absence of a control group. Observed changes may partly reflect non‑specific influences such as expectancy effects, regression to the mean, or natural symptom fluctuation. Although fatigue in primary care typically shows a persistent course with limited spontaneous improvement, and care-as-usual groups in prior CBT trials generally report smaller gains than structured interventions [9, 10, 18], definite conclusion regarding efficacy cannot be drawn without conducting a well-powered RCT.

While no mediation analyses were conducted, the patterns of change in fatigue severity and CBRQ sub-scales is consistent with findings from previous trials of disorder-specific CBT for fatigue [13], and merits formal mediation analysis within the framework of a randomized trial.

### Strengths and limitations

Strengths of this study include its delivery in routine primary care (enhancing ecological validity), the transdiagnostic inclusion criteria, and minimal data attrition. Limitations include the small sample size, non-randomized design, absence of follow-up data, and in-depth post-treatment interviews. These limitations are expected in an initial feasibility study but preclude conclusions about effectiveness. In addition, while several exclusion criteria were applied to ensure participant safety (e.g., severe psychiatric disorders, suicide risk, and substance use) and address practical requirements of the digital format (e.g., language proficiency and internet access), these eligibility restrictions limit the representativeness of the sample. The predominantly female sample further restricts generalizability to other genders. The authors also acknowledge that the site where the study was conducted, Gustavsberg PCC, is a large clinic with established psychological treatment infrastructure, which may limit the generalizability of feasibility to smaller and less specialized primary care settings.

The intervention included gradual increases in physical activity introduced in small, individualized increments and was not strictly symptom contingent. Progression was collaboratively negotiated and flexibly adjusted, rather than delivered as a fixed incremental schedule. Of note, however, the intervention differs from parts of the NICE guidance for ME/CFS [35] in that symptom fluctuations were not used as the primary determinant of activity progression. While many participants reported improvement, and an individual patient data meta-analysis found no indication that ME/CFS patients who reported PEM benefitted less from CBT [36], this feasibility study does not allow conclusions regarding safety or effectiveness for individuals with ME/CFS or PEM specifically. Accordingly, the findings should be interpreted as informing of feasibility and acceptability only.

### Implications for intervention refinement and future evaluation

If effectiveness is confirmed, tCBT for fatigue could be easier to implement than multiple disorder-specific protocols, as therapists would only need to be trained in one therapy, potentially improving scalability and access in primary care. Based on the results of this feasibility study, some possible refinements to the treatment and safety routines have been identified. These include (1) optional modules to allow for greater individual tailoring; (2) increased therapist–patient contact in later stages to sustain adherence; and (3) continuous monitoring of adverse effects during treatment, rather than solely post-treatment, to capture their timing and resolution more precisely. These refinements will inform the design of a subsequent RCT to evaluate the efficacy of tCBT.

## Conclusion

In summary, this study provides preliminary support for the feasibility and acceptability of delivering tCBT for severe and persistent fatigue in routine primary care. While these findings are encouraging, the single-arm design does not allow conclusions regarding effectiveness, which motivates further evaluation within the framework of an RCT. If effectiveness is established in future works, a transdiagnostic approach may offer a scalable intervention to improve access to evidence-based treatment for patients with severe and persistent fatigue in primary care.

## Supplementary Information

Supplementary Material 1

## Data Availability

The datasets used during the current study are available from the corresponding author on reasonable request.

## Abbreviations

CBT: Cognitive behavioral therapy
tCBT: Transdiagnostic Cognitive behavioral therapy
RCT: Randomized controlled trial
PCC: Primary Care Center
GP: General practitioner
PEM: Post-exertional malaise
C-scale: Credibility/Expectancy Questionnaire
CSQ: 8-Client Satisfaction Questionnaire
WAI: Working Alliance Inventory
NEQ: 20-The Negative Effects of Treatment Questionnaire
CIS: Checklist Individual Strength
CIS: F-Checklist Individual Strength-Fatigue severity subscale
WSAS: Work and Social Adjustment Scale
CBRQ: Cognitive and Behavioral Responses to Symptoms Questionnaire
IQR: Interquartile Range
SD: Standard deviation
