# Supplementary material for: Transdiagnostic cognitive behavioral therapy for severe and persistent fatigue – a feasibility study in primary care
Source: BMC Prim Care. 2026 Apr 29;27:168. doi: 10.1186/s12875-026-03346-x (PMC13130435; doi:10.1186/s12875-026-03346-x)
Supplement: Supplementary file 1 — Supplementary Material 1 [file 12875_2026_3346_MOESM1_ESM.pdf]

Supplementary material

Transdiagnostic cognitive behavioral therapy for severe and persistent fatigue – a feasibility study in primary care

Frank Svärdman[ab*], Conrad Samuelsson[ab], Ludwig Franke-Föyen[abcd], Anna Oremark[b], Anna Högfeldt, Jacob Andersson Emad[bf], Douglas Sjöwall[f], Christian Rück[g], Erik Hedman-Lagerlöf[ab], Hans Knoop[ah], Elin Lindsäter[abg].

[a]Division of Psychology, Department of Clinical Neuroscience, Karolinska Institutet, Stockholm, Sweden
[b]Gustavsberg University Primary Care Center, Academic Primary Care Center, Region Stockholm and Karolinska Institutet
[c]Stress Research Institute, Department of Psychology, Stockholm University, Stockholm, Sweden
[d]Osher Center for Integrative Health, Department of Clinical Neuroscience, Karolinska Institutet, Stockholm, Sweden
[e]Family Medicine and Primary Care, Department of Neurobiology, Care Sciences and Society, Karolinska Institutet
[f]Center for Neurodevelopmental Disorders at Karolinska Institutet (KIND), CAP Research Center, Region Stockholm, Sweden
[g]Centre for Psychiatry Research, Department of Clinical Neuroscience, Karolinska Institutet and Stockholm Healthcare Services, Stockholm, Sweden
[h]Departement of Medical Psychology, Amsterdam University Medical Centre and Amsterdam Public Health Research Institute, University of Amsterdam, Amsterdam, The Netherlands

*Corresponding author. E-mail: frank.svardman@ki.se

Transdiagnostic cognitive behavioral therapy for severe and persistent fatigue–a feasibility study in primary care

Supplementary material 1

Additional baseline variables 3
Diagnostic Criteria for exhaustion disorder 4
Self-reported believed cause of fatigue 4
Definitions of activity disability levels 5
Assessment of Post exertional malaise (PEM) 5
Symptoms of Myalgic encephalomyelitis/Chronic fatigue syndrome (ME/CFS) 6
Treatment description and overview 7
Internet Modules activated and patients' engagement 10
Participant adherence to treatment 10
Therapist fidelity to treatment 11
Fidelity rating instrument 11
Negative effects 13
NEQ-20 free-text answers 14
Questionnaire regarding therapist's feedback on the treatment 15
Qualitative analysis of therapist's feedback on the treatment 15
Participants feedback on the treatment 17
Exploratory effectiveness outcomes 19
Descriptive statistics of additional clinical outcomes 20
Process measures (every third week) 21
Graphs CIS-F and CBRQ: Process measures (every third week) 22
CONSORT Checklist for pilot or feasibility trial 23
References 26

Additional baseline variables

The additional baseline variables were collected during the online-screening and in clinical assessments by a general practitioner and a licensed psychologist.

Table S1. Additional baseline variables collected at screening or clinical assessment (N=18)

	n (%)
**Country of Birth**	
Sweden	16 (89)
Other	2 (11)
**Have Children**	
Yes	14 (78)
No	4 (22)
**Living Situation**	
Single	3 (17)
Single + children	4 (22)
Partner + children	10 (56)
Partner, no children	1 (6)
**Sickness Benefit/Welfare Support**	
No sickness benefit or welfare support	5 (28)
Sickness benefit	9 (50)
Financial assistance	3 (17)
Activity support[1]	1 (6)
**Comorbid somatic symptoms and disorders[2]**	
Tension headache	11 (61)
Allergy	10 (56)
Asthma	6 (33)
Migraine	6 (33)
Tinnitus	6 (33)
High blood pressure	4 (22)
Chronic back pain	3 (17)
IBS	3 (17)
POTS (Postural Orthostatic Tachycardia Syndrome)	2 (11)
Thyroid disease	2 (11)
Gynecological disease, e.g., endometriosis, PCOS	2 (11)
Ehlers-Danlos Syndrome/Hypermobility Syndrome	2 (11)
Dyspepsia	2 (11)
Major depression	2 (11)
Anxiety disorders	2 (11)
Celiac disease	1 (6)
Atopic eczema	1 (6)
Palpitations without diagnosis	1 (6)
ADHD	1 (6)
**Alcohol Use Disorders Identification (AUDIT)**	
Mean (SD)	2·38 (2·06)
Range	2-7
**Current psychopharmacological treatment[2]**	
Antidepressive drugs	7 (39)
Sleep medicine/sedative	6 (33)
**Previous self-reported treatments**	
Psychopharmacological	3 (17)
Physiotherapy	3 (17)
Psychologist CBT	3 (17)
Psychologist not CBT	2 (11)
Alternative medicine	1 (6)
Acupuncture	3 (17)
Massage therapy	0 (0)
Multimodal rehabilitation	3 (17)
Other[3]	4 (22)
**Severe adverse life event the year before fatigue onset**	4 (22)
**Ongoing infection at fatigue onset**	
COVID-19	4 (22)
Other infection	5 (28)

**Met criteria for Exhaustion disorder[4]**	4 (22)

Note. PCOS, Polycystic Ovary Syndrome. [1] Activity support is a financial benefit provided by the Swedish Social Insurance Agency to individuals who are participating in labor market programs organized by the Swedish Public Employment Service. [2] Reported during clinical assessments by a general practitioner. [3] Examples given: massage, osteopathy, occupational therapy, yoga. [4] Based on clinical assessment using diagnostic criteria for exhaustion disorder as presented in Table S2.

Diagnostic Criteria for exhaustion disorder

Table S2. Diagnostic Criteria for exhaustion disorder (F43.8A, ICD10-SE).

A. Physical and mental symptoms of exhaustion during at least 2 weeks. The symptoms have developed in response to one or more identifiable stressors, which have been present for at least 6 months.

B. Markedly reduced mental energy, manifested by reduced initiative, lack of endurance, or increased time needed for recovery after mental efforts.

C. At least four of the following symptoms have been present most of the day, nearly every day, during the same 2-week period:

1. Persistent complaints of impaired memory and concentration.
2. Markedly reduced capacity to tolerate demands or to perform under time pressure.
3. Emotional instability or irritability.
4. Insomnia or hypersomnia.
5. Persistent complaints of physical fatigue and lack of endurance.
6. Physical symptoms such as muscular pain, chest pain, palpitations, gastrointestinal problems, vertigo, or increased sensitivity to sounds.

D. The symptoms cause clinically significant distress or impairment in social, occupational, or other important areas of functioning.

E. The symptoms are not due to the direct physiological effects of a substance (e.g., abuse of a drug or medication) or a general medical condition (e.g., hypothyroidism, diabetes, infectious disease).

F. If criteria for major depression, dysthymia, or generalized anxiety disorder are met simultaneously, exhaustion disorder is set as an additional specification to any such diagnosis.

Note: All criteria with capital letters must be met to set the diagnosis. Criteria correspond to those published by the Swedish National Board of Health and Welfare in 2003

Self-reported believed cause of fatigue

Participants reported their believed cause of fatigue during the screening procedure, all answers are presented in Table S3. It was possible to choose multiple causes, eight participants (44%) reported three or more causes.

Table S3. Patient-reported perceived cause of fatigue (N=18)

What do you believe caused your fatigue?	n (%)
Stress or anxiety	8 (44)
Heredity – it runs in my family	2 (11)
Traumatic events during childhood	2 (11)
A bacterium or a virus	9 (50)
Diet or eating habits	1 (6)
Coincidence or bad luck	1 (6)
Poor previous medical care	0 (0)
Environmental pollution	0 (0)
Allergies	0 (0)
Family problems or concerns in close relationships	4 (22)

Too much work	3 (17)
Aging	0 (0)
Perimenopause or menopause	4 (22)
My lifestyle habits (e.g., alcohol, smoking, sedentary lifestyle)	0 (0)
Accident or injury (e.g., head trauma, concussion)	1 (6)
Cancer or cancer treatment	2 (11)
Hormonal disease (e.g., diabetes, thyroid disease)	1 (6)
Infectious disease (e.g., mononucleosis, pneumonia, sepsis, Lyme disease)	1 (6)
Chronic joint or muscle pain (e.g., fibromyalgia, pain after whiplash injury)	1 (6)
Rheumatic disease (e.g., rheumatoid arthritis, psoriatic arthritis)	0 (0)
Neurological disease (e.g., Parkinson's disease, multiple sclerosis, stroke)	1 (6)
Heart disease (e.g., heart failure, high blood pressure)	0 (0)
Gynecological disease (e.g., endometriosis)	0 (0)
Psychiatric disease (e.g., depression, anxiety disorders, PTSD, ADHD, ASD)	2 (11)
My personality	1 (6)
Changes in the immune system	2 (11)

Note: PTSD, Post-Traumatic Stress Disorder; ADHD, Attention-Deficit Hyperactivity Disorder; ASD, Autism Spectrum Disorder

Definitions of activity disability levels

During the psychiatric clinical assessment done by the licensed psychologist, an overall activity disability level was assessed in accordance with the definitions provided below. The answers have been translated to English partly using Chat GPT, version 4o and Microsoft Copilot using the prompt "*Translate the following text to English*".

1. **Mild** (functional capacity is reduced to half compared to before the illness; one can work, study part-time, and do household chores, but this comes at the expense of socializing and engaging in leisure activities. Weekends are needed for recovery)
2. **Moderate** (barely able to leave the house, and often needs to rest for several hours during the day)
3. **Severe** (bedridden for most of the day. Only able to perform light activities such as brushing teeth and eating. Many experience serious cognitive difficulties)
4. **Very severe** (completely bedridden around the clock. Needs help with all daily tasks. In the most extreme cases, any form of interaction or sensory input is an overwhelming burden)

Assessment of Post exertional malaise (PEM)

Post exertional malaise (PEM) was clinically assessed by a licensed psychologist, based on participant responses to questions in Table S4. PEM was defined as worsening of symptoms and functional ability after a minor exertion with symptoms lasting 24 hours or more.

Table S4. Assessment of Post exertional malaise (PEM) (N=18)

	n (%)
Do your symptoms worsen after exertion, and if so, does the deterioration persist for at least 24 hours after the exertion?	
**Yes, for at least 24 hours**	13 (72)
To some extent (less than 24 hours)	5 (28)
Do you experience any of the the following symptoms after exertion?	
Flu-like symptoms / fever / feeling feverish	7 (39)
Muscle weakness	9 (50)

Pain	6 (33)
Sore throat	4 (22)
Concentration difficulties	13 (72)
Brain fog	12 (67)
Cardiac symptoms	3 (17)

Note: PEM was assessed by a licensed psychologist

Symptoms of Myalgic encephalomyelitis/Chronic fatigue syndrome (ME/CFS)

Symptoms commonly associated with ME/CFS were rated by the assessing licensed psychologist based on the symptom ratings displayed in Table S5. Patients were asked to report symptoms they had experienced during the last two weeks.

Table S5. Clinician-assessed ME/CFS Symptoms (N=18)

**General pain**	**n (%)**
Mild	1 (6)
Moderate	3 (17)
Severe	1 (6)
**Joint pain**	
Mild	3 (17)
Moderate	3 (17)
**Morning stiffness**	
Mild	6 (33)
Moderate	2 (11)
Severe	1 (6)
Unbearable	1 (6)
**Headache**	
Mild	3 (17)
Moderate	4 (22)
Severe	3 (17)
**Fatigue**	
Moderate	6 (33)
Severe	12 (67)
Unbearable	1 (6)
**Body tenderness**	
Mild	3 (17)
Moderate	3 (17)
Severe	1 (6)
**Dizziness**	
Mild	6 (33)
Moderate	4 (22)
Severe	1 (6)
**Irritable bowel**	
Mild	2 (11)
Moderate	6 (33)
Severe	1 (6)
**Overactive bladder**	
Moderate	3 (17)
**Restless legs**	
Mild	1 (6)
Moderate	2 (11)
Severe	1 (6)
**Memory difficulties**	
Mild	2 (11)
Moderate	11 (61)
Severe	3 (17)
Unbearable	1 (6)
**Concentration difficulties**	
Mild	5 (28)
Moderate	8 (44)
Severe	2 (11)
Unbearable	1 (6)
**Sleep disturbances**	
Mild	2 (11)
Moderate	5 (28)
Severe	4 (22)
**Chills/sweating**	
Mild	1 (6)

Moderate	4 (22)
Severe	1 (6)
Unbearable	1 (6)
**Numbness/paresthesia**	
Mild	3 (17)
Moderate	2 (11)
Severe	1 (6)
**Palpitations**	
Mild	4 (22)
Moderate	4 (22)
**Fever**	
Mild	1 (6)
Moderate	2 (11)
**Tender lymph nodes in the neck or armpits**	
Unbearable	1 (6)

Note: All symptoms are rated on the following scale: No, Mild, Moderate, Severe, and Unbearable. Empty response options have been removed in the table.

Treatment description and overview

An overview of transdiagnostic Cognitive behavior therapy (tCBT) during all weeks of treatment and corresponding internet modules is presented in Figure S1. It should be noted that the overview is an approximant model and adaptions from the outlined timepoints could be made depending on the characteristics of the patients and progress in treatment.

Optional modules could be activated when therapists deemed it appropriate, the following optional modules were available: (1) Anxiety and worry (2) Stress (3) Pain (4) To live with chronic disease (5) Mental activity (6) Return to work, and (7) Social activity.

Description of treatment phases

- Phase 1: Face-to-face sessions were booked weekly or bi-weekly. The online platform primarily provided psychoeducation and worksheets, including information about fatigue, goal setting, sleep-wake regulation, attention shifting, and cognitive restructuring (and evening out activity when appropriate).
- Phase 2: Face-to-face sessions were less frequent, with increased use of the online platform for asynchronous therapist-participant communication. The main component of phase 2 was gradual activity increase, beginning with physical activity (e.g., walking, cycling) and later progressing to increasing mental and social activities.
- Phase 3: Most of the participant-therapist contact occurred via the digital platform, but synchronous sessions could be booked based in participant needs. Phase 3 focused on goal attainment, aiming at realizing goals and no longer being hindered by severe fatigue.

Figure S1. Overview of tCBT treatment structure and content

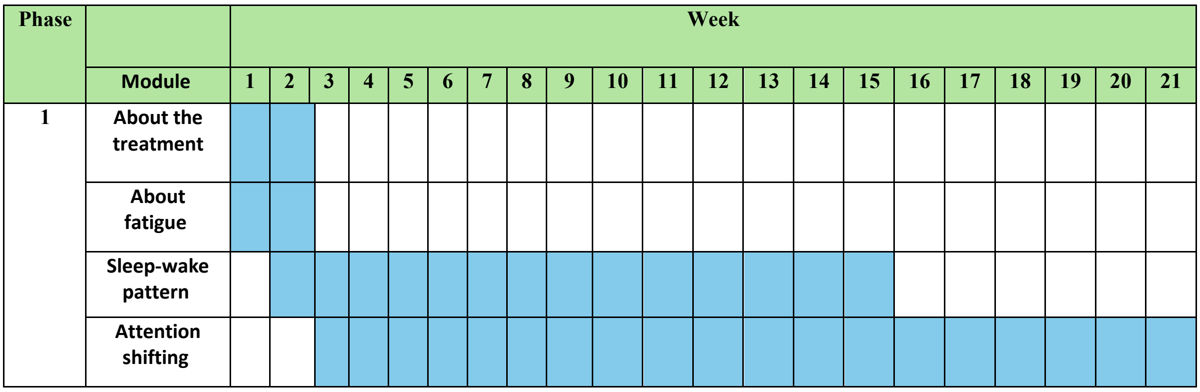

	Unhelpful thoughts																					
	Uneven activity*																					
2	Gradual activity increase																					
3	Goal realization																					
	Maintaining progress																					

*Only participants that have an uneven activity pattern at baseline

Table S6. The TIDieR (Template for Intervention Description and Replication) Checklist: Description of *transdiagnostic Cognitive behavior therapy (tCBT) for fatigue delivered in a blended format*

Item number	Item	Other † (details)
1.	**BRIEF NAME**<br>*Transdiagnostic Cognitive behavior therapy (tCBT) for fatigue*	
2.	**WHY**<br>tCBT is a transdiagnostic blended cognitive behavioral treatment for persistent fatigue, based on the biopsychosocial model and cognitive-behavioral theory. It addresses cognitive and behavioral maintenance factors of fatigue.	
3.	**WHAT**<br>tCBT is delivered using synchronous on-site or videoconference sessions and using asynchronous online material and communication. Materials include psychoeducational text and CBT exercises accessible via the digital platform BASS4 delivered using online "modules". The main modules are "About the treatment", "About fatigue", "Sleep-wake pattern", "Attention shifting", "Unhelpful thoughts", "Uneven activity (only for participants with an uneven activity pattern), "Gradual activity increase", "Goal realization", and "Maintaining progress" and optional modules are related to the following topics: "Anxiety/Worry", "Stress", "Pain", "Living with chronic illness", "Mental activity", "Return to work", and "Social activity".<br>Therapists use session report cards to log the format, content and duration of each session.	
4.	1. **Initial phase – Psychoeducation and activity stabilisation:**<br>Participants receive psychoeducation about fatigue from a biopsychosocial framework. The psychoeducative approach differentiates the factors involved in fatigue onset (e.g., an infection, a stroke, prolonged work life stress without adequate recovery), and factors currently maintaining it (e.g., a disturbed sleep-wake pattern, a boom-and-bust activity pattern, unhelpful beliefs about symptoms, symptom focusing). Participants and therapists collaboratively identify cognitive and behavioural factors contributing to the maintenance of the fatigue. This phase involves individual goal setting, stabilisation of the sleep-wake cycle, evenly distributing daily activities, as well as training in attention-shifting and cognitive reappraisal techniques. The participant is invited to include their partner, parents or other close relation at one or more sessions in this phase. Participants usually have weekly 45-minute synchronous sessions during phase 1 which will last between 3-6 sessions depending on the participant's need.<br>2. **Second phase – Gradual activity increase:**<br>Participants gradually increase their activity level. Participants make a schedule for	

gradually increasing their activity without depending on fatigue level. Initially, physical activity is gradually increased (e.g., walking or cycling). Subsequently, participants work on gradually increasing their mental and social activity levels as needed to accomplish goals. Participants and therapists mainly communicate with short synchronous video-based sessions or using the message function of the digital platform.

3. **Final phase – Goal attainment:**
Having succeeded in gradually increasing their activity to a desired level, participants actively pursue their individually formulated goals. The overarching objective is for participants to no longer experience significant functional limitations due to fatigue. Participants return to an activity schedule that is no longer evenly distributed. Contact with the therapist is highly individualised depending on the needs of the participant.

WHO PROVIDED

5. Three Licensed psychologists and one residential psychologist employed at Gustavsberg University Primary Care Centre with experience in CBT in primary care. They receive a two-day training workshop before the trial and bi-weekly supervision from an expert clinician.

HOW

6. Blended format combining synchronous individual face-to-face or video-based sessions and asynchronous individual therapist-patient communication via the BASS digital platform.

WHERE

7. On-site at participating primary care centers or remotely via video and the digital platform.

WHEN and HOW MUCH

8. Duration: 4–6 months; total sessions: 5–15 (at least one face-to-face); synchronous sessions usually 45 minutes, with weekly sessions during Phase 1 (3–6 weeks). Contact in later phases is individualized.

TAILORING

9. The tCBT protocol can be tailored to suit participants needs and is done so in collaboration with the participant. The number of synchronous sessions and their modality (face-to-face or video-based) depends on the participant's needs. The inclusion of a close relation is optional. The session content and order are dependent on the needs of the participant. However, tCBT is defined to last between 4-6 months and to encompass between 5 and 15 sessions, with at least five session being face-to-face, and always includes the following modules: psychoeducation about fatigue, goal setting, unhelpful thoughts about symptoms, symptom focusing, gradual increase of activity, and goal fulfilment. Sleep-wake pattern is addressed with all participants who do not have a stable sleep-wake pattern and/or who report daytime sleep, and even distribution of activity is addressed with all participants who report an uneven activity pattern (referring to having periods of increased activity followed by an increased period of inactivity). Several optional modules can be delivered based on the participant's needs: "Anxiety/Worry", "Stress", "Pain", "Living with chronic illness", "Mental activity", "Return to work", and "Social activity".

MODIFICATIONS

10.[ǂ] No modifications to the intervention were made during the trial.

HOW WELL

11. Planned: Therapists will participate in a two-day training session before initiating treatments within the trial. Therapists will receive supervision from a professor with expertise in the treatment manual every other week.

To evaluate fidelity, all synchronous sessions will be audio recorded, and a fidelity scale has been developed. A satisfactory session is defined as a mean score of ≥3. To ensure tCBT is delivered according to protocol, therapists will complete session report cards for each therapy session, indicating current phase of treatment, which specific interventions were administered, if a close relation was present, as well as modality (face-to-face, video) and length of the session.

	To evaluate participants adherence to treatment, participants and therapist separately rated participant adherence at the post treatment measurement point.
**12.**[‡]	Actual: The overall fidelity was satisfactory (mean 3·6, SD 0·5), and 93% of sessions were rated as having satisfactory fidelity (≥3 or above on all domains).<br><br>Therapists reported high or complete adherence for 12 (67%) participants in phase one, seven (39%) in phase two, and four (22%) in phase three. According to participants' own ratings, 15 (83%) reported high or complete adherence in the first half of treatment, and eight (44%) in the second half.

Internet Modules activated and patients' engagement

Table S7 displays the number of modules that were activated to participants by therapist and the number of modules that participants actively with engaged (reading the informative text and/or did the assignments provided in the module). It should be noted that this however does not fully correspond to participants engaging in the theme of the internet module. Participants received information and exercises in face-to-face sessions and the internet material was often used as a reminder while also giving participants a structured way to register assignments. This means that it was possible to do the exercises but not engage with the internet module.

Table S7. Internet Modules activated and engaged by participants

**Mandatory modules**	Activated by therapist	Number of participants engaging
About the treatment	18	18
About fatigue	18	16
Sleep-wake pattern	18	16
Attention shifting	18	15
Unhelpful thoughts	17	11
Uneven activity*	16	11
Gradual activity increase	17	15
Goal realization	14	10
Maintaining progress	12	9
**Optional modules**		
Anxiety/Worry	4	4
Stress	1	1
Pain	2	2
Living with chronic illness	0	0
Mental activity	4	1
Return to work	2	0
Social activity	3	1

*Only participants that have an uneven activity pattern at baseline. *N* = 18

Participant adherence to treatment

At the post assessment, assessments of adherence to treatment were rated both by therapists and participants (self-assessments).

Therapist and participants rated adherence on a five-point scale ("*Not at all", "Small extent", Moderate extent", "Large extent" and "Completely"*). However, therapist rated participants dived on phase of treatment, while participants were asked to rate their adherence divided into the first and then second half of the treatment.

Table S8 shows the proportion of "Largely" and "Complete" adherence answers by therapist and participants.

Table S8. Participants adherence to treatment reported by therapists and participants (N=18)

**Therapist assessment of patient adherence**	n (%)
Largely or completely in phase 1	12 (67)

Largely or completely in phase 2	7 (39)
Largely or completely in phase 3	4 (22)
**Participants assessment of own adherence**	
Largely or completely in first half	15 (83)
Largely or completely in second half	8 (44)

Therapist fidelity to treatment

Assessment of treatment fidelity was based on audio-recorded therapy sessions of which a randomly selected sample was rated using a 7-item scoring instrument, using a 5-point Likert Scale (range, "Not adherent" to Fully adherent") on each item. In cases where the specific item was not applicable to the session in question, a "Not Applicable" option was available. The instrument was developed by the research team (see page 6 for scoring instrument). The fidelity instrument was developed by drawing inspiration from established fidelity instruments including the *The cognitive therapy adherence and competence scale* (C-TACS) (1), and the *Dialectical behavior Therapy Adherence Coding Scale* (DBT ACS) (2).The procedure for data collection and analysis was inspired by the Okkersen and colleagues (3), which investigated treatment adherence and fidelity in a study of CBT for severe fatigue in patients with myotonic dystrophy.

The total number of synchronous sessions conducted was 189, of which 171 (90·5%) (retention due to technical issues or oversight) were recorded. Of these, 46 (26·9% of recorded sessions) were removed due to being conducted by therapist who conducted the rating. The total number of sessions retained for sampling was then 125 (73·1% of recorded sessions), of which 15 (8·7% of recorded sessions) were sampled for fidelity rating.

The total mean therapist's fidelity score was 3·6 (min-max, 2·5-4·5). The proportions of items rated as "not applicable" was 25 out of 105 (23·8%). Table S9 displays average fidelity in each fidelity variable.

Table S9. Assessment of therapist fidelity to treatment

Fidelity aspect	Mean score (scale range 1 to 5)
1. Treatment content	4·2
2. Activity registration	3·6
3. Reference to the patient's goals	3·2
4. Orienting focus from fatigue	3·6
5. Helpful and unhelpful thoughts	2·9
6. Sharing responsibility	3·7
7. Eliciting feedback from the patient	4·0

Fidelity rating instrument

Table S10. Fidelity rating criteria

1. **Treatment content.**

5 – Fully adherent. Session content is in accordance with the session report card, *and*
Treatment content is delivered in accordance with the tCBT manual, covering the most important points of the manual. The session is mainly on target, spending only a little time on matters not regarding key treatment interventions.

4 – Mostly Adherent.

3 – Moderately Adherent. Session content is in accordance with the session report card, *but*
treatment interventions are not delivered in sufficient accordance with the CFS manual, covering some but not most of the important points of the manual, and/or spends a moderate amount of time off target.

2 – Somewhat Adherent

1 – Not adherent. Session content is not in accordance with the session report card, *and/or*

Treatment content markedly departs from the CFS manual, failing to cover several important points indicated in the manual, <u>*and/or*</u>
the therapist spends substantial amounts of time off target.

2. Activity Registration

5 – Fully adherent. The activity registration is explicitly and thoroughly reviewed with the patient. If activity was not properly recorded, or the recorded activity did not follow the graded activity pattern, the therapist put sufficient time into problem-solving with the patient in a helpful manner, <u>*or*</u>
The patient is still in Phase 1 and has not begun with activity registration, or is in Phase 3 and does not require further activity registration.

4 – Mostly Adherent.

3 – Moderately Adherent. Reviewing the activity registration is appropriate at the current stage of treatment, <u>*and*</u>
The therapist reviews the activity registration but does not respond to improperly recorded or non-adherent registration in a sufficiently skilled or helpful manner.

2 – Somewhat Adherent

1 – Not adherent. Reviewing the activity registration is appropriate at the current stage of treatment, <u>*and*</u>
The therapist does not review the activity registration card, or the therapist reviews the activity registration card but does not respond to improperly recorded registration, or does so in a judgmental or unhelpful manner.

3. Reference to the patient's goals

5 – Fully adherent. The therapist consistently discusses treatment rationale and progress in terms of the patient's ability to approach or achieve their goals, not in terms of fatigue reduction. The therapist consistently uses activity-oriented phrases such as "Given your goals, how can you do [intervention]", "What can you do now, that you couldn't do at the start of treatment", and "Will it help with your goals?"

4 – Mostly Adherent.

3 – Moderately Adherent. The therapist sometimes refers to the patient's goals when providing rationale for interventions or when discussing treatment progress, but does not consistently use activity-oriented phrases, sometimes discussing the treatment in terms of fatigue reduction or without any reference to the patient's goals.

2 – Somewhat Adherent

1 – Not adherent. The therapist does not refer to the patient's goals or misses several opportunities to link the treatment interventions to patient goals. The patient makes unhelpful statements about treatment progress in terms of having fatigue and the therapist does not utilize activity-oriented phrases.

4. Orienting focus from fatigue

5 – The therapist is attentive to the patient referring to their symptoms during the session, and encourages the patient to shift focus away from symptoms by providing a rationale in collaboration with the patient <u>*or*</u>
The patient does not refer to their symptoms during the session, <u>*or*</u>
The treatment is in Phase 1 and it is not yet appropriate to address focus on symptoms (i.e. the session report card shows that attention on symptoms has not yet been addressed).

4 – Mostly Adherent.

3 – Moderately Adherent. The therapist notices that the patient is focusing on symptoms, but does not sufficiently address it with the patient in an explorative and collaborative manner, e.g. "*I noticed that you talked about fatigue there, that will only make you more fatigued".*

2 – Somewhat Adherent

1 – Not adherent. The session report card indicates that addressing focus on symptoms is appropriate (i.e. focus on symptoms has been addressed in a previous session), <u>*and*</u>
The patient refers to their symptoms in an unhelpful way during the session and the therapist does not address focus on fatigue or does so in a judgmental and/or unhelpful manner.

5. Helpful and unhelpful thoughts

5 – The therapist identifies unhelpful thoughts about fatigue* or other symptoms and discusses the maintaining effect of such thoughts with the patient, helping the patient verbalize more helpful thoughts, <u>*or*</u>
The patient does not express any unhelpful thoughts about fatigue during the session.

4 – Mostly Adherent.

3 – Moderately Adherent. The patient expresses unhelpful thought about fatigue during the session, and the therapist addresses the thought but does not help the patient verbalize more helpful thoughts.

2 – Somewhat Adherent

1 – Not adherent. The patient expresses unhelpful thoughts about fatigue during the session, and the therapist does not address the thoughts or does so in a non-helpful manner, e.g. by not using Socratic questions or by using judgmental or prescriptive language.

6. Sharing responsibility

5 – Fully adherent. The therapist actively encourages the patient to take an active role in their recovery and share responsibility for all aspect on the treatment, asking questions such as "Would you like to know what I think?", "What do you think you need to do to succeed with [the intervention]?", or "How do *you* want to proceed?".

4 – Mostly Adherent.

3 – Moderately Adherent. The therapist sometimes encourages the patient to take responsibility for aspects of the treatment, but does not do so consistently, and does not leave sufficient time for the patient to reach their own conclusions.

2 – Somewhat Adherent

1 – Not adherent. The therapist makes little or no attempt to involve the patient in the treatment process, telling the patient what to do without leaving space for the patient to reach their own conclusions. Treatment – as – prescription.

7. **Eliciting feedback from the patient**

5 – Fully adherent. The therapist consistently checks the patient's understanding of and reaction to important aspects of the session (e.g. the psychoeducation about CBT or maintaining factors, planned interventions) using open questions to elicit the patients views and reactions to the session (e.g. "What is your reaction to what I just said", "As you see it, why is it important to do [the intervention]?", or "Do you have concerns about what we spoke about today?", *or*
It is clear from context that asking for the patient's feedback is superfluous (e.g. because the session is merely a "check-in" during gradual activity increase and no problems have arisen).

4 – Mostly Adherent.

3 – Moderately Adherent. The therapist occasionally checks the patient's understanding of important aspects of the session,, but does not do so consistently or does not leave sufficient space for the patient to respond.

2 – Somewhat Adherent

1 – Not adherent. The therapist makes little or no effort to discuss the patient's understanding of and reaction to the session content when appropriate, *or*
Ignored such feedback from the patient.

*By unhelpful thoughts, particular importance is given to the putative maintaining thoughts targeted in the fatigue treatment, such as damage beliefs, embarrassment beliefs, or non-acceptance of symptoms.

Negative effects

Table S11 displays a full report of negative effects as reported by the t*he Negative Effects of Treatment Questionnaire (NEQ-20)* dived into total scores on every item, number of effects reported as moderately affected or higher, and if effects were attributed to treatment. The overall questions for every item are as follows: "Did you experience this: *Yes* or *No*?", "If yes – here is how negatively it affected me: *Not at all, Slightly, Moderately, Very*, *Extremely*", and "Probably caused by: "*The treatment I received"* or "*Other circumstances".*

It should be noted that NEQ asks for negative effects during the treatment phase, not after treatment.

Table S11. Results of the Negative Effects of Treatment Questionnaire (NEQ-20).

Item #		n (%)
1	**I had more problems with my sleep**	7 (39)
	I had more problems with my sleep - Moderately affected or higher	4 (22)
	I had more problems with my sleep - Due to treatment	3 (17)
2	**I felt like I was under more stress**	6 (33)
	I felt like I was under more stress - Moderately affected or higher	4 (22)
	I felt like I was under more stress - Due to treatment	2 (11)
3	**I experienced more anxiety**	4 (22)
	I experienced more anxiety - Moderately affected or higher	3 (17)
	I experienced more anxiety - Due to treatment	2 (11)
4	**I felt more worried**	4 (22)
	I felt more worried - Moderately affected or higher	3 (17)
	I felt more worried - Due to treatment	1 (6)
5	**I experienced more hopelessness**	4 (22)
	I experienced more hopelessness - Moderately affected or higher	3 (17)
	I experienced more hopelessness - Due to treatment	2 (11)
6	**I experienced more unpleasant feelings**	5 (28)
	I experienced more unpleasant feelings - Moderately affected or higher	5 (28)
	I experienced more unpleasant feelings - Due to treatment	3 (17)
7	**I felt that the issue I was looking for help with got worse**	4 (22)

	I felt that the issue I was looking for help with got worse - Moderately affected or higher	4 (22)
	I felt that the issue I was looking for help with got worse - Due to treatment	4 (22)
**8**	**Unpleasant memories resurfaced**	3 (17)
	Unpleasant memories resurfaced - Moderately affected or higher	1 (6)
	Unpleasant memories resurfaced - Due to treatment	0 (0)
**9**	**I became afraid that other people would find out about my treatment**	0 (0)
**10**	**I got thoughts that it would be better if I did not exist anymore and that I should take my own life**	1 (6)
	I got thoughts that it would be better if I did not exist anymore and that I should take my own life - Moderately affected or higher	1 (6)
	I got thoughts that it would be better if I did not exist anymore and that I should take my own life - Due to treatment	1 (6)
**11**	**I started feeling ashamed in front of other people because I was having treatment**	0 (0)
**12**	**I stopped thinking that things could get better**	2 (11)
	I stopped thinking that things could get better - Moderately affected or higher	2 (11)
	I stopped thinking that things could get better - Due to treatment	1 (6)
**13**	**I started thinking that the issue I was seeking help for could not be made any better**	3 (17)
	I started thinking that the issue I was seeking help for could not be made any better - Moderately affected or higher	2 (11)
	I started thinking that the issue I was seeking help for could not be made any better - Due to treatment	1 (6)
**14**	**I think that I have developed a dependency on my treatment**	0 (0)
**15**	**I did not always understand my treatment**	1 (6)
	I did not always understand my treatment - Moderately affected or higher	0 (0)
**16**	**I did not always understand my therapist**	1 (6)
	I did not always understand my therapist - Moderately affected or higher	0 (0)
	I did not always understand my therapist - Due to treatment	0 (0)
**17**	**I did not have confidence in my treatment**	3 (17)
	I did not have confidence in my treatment - Moderately affected or higher	2 (11)
	I did not have confidence in my treatment - Due to treatment	1 (6)
**18**	**I felt that the treatment did not produce any results**	3 (17)
	I felt that the treatment did not produce any results - Moderately affected or higher	2 (11)
	I felt that the treatment did not produce any results - Due to treatment	2 (11)
**19**	**I felt that my expectations for the therapist were not fulfilled**	0 (0)
**20**	**I felt that the treatment was not motivating**	0 (0)

Note: N = 18

NEQ-20 free-text answers

Eight participants provided free-text answers in NEQ to the question "*Describe in your own words if there were any other negative events and effects, and what characterized them*". The answers have been translated to English partly using Chat GPT, version 4o and Microsoft Copilot using the prompt "*Translate the following text to English*".

Participant 1

"I experienced more anxiety at times, but it was related to working hard on my problem areas. Overall, I feel better even regarding anxiety, but in different parts of the treatment, it required me to work through some things to move forward. As for reliving difficult memories, it is connected to gaining a better understanding of myself by looking at events from a new perspective. It can be tough in the process, but moving forward, I see greater opportunities to live well by understanding how I react to things "

Participant 2

"The negative effects arose because I felt my head could start thinking again in small and moderate doses before the mental fatigue sets in. I can think and see my situation more clearly and then become updated on my

financial situation and my ability to work, which makes me worried and sad - I want more than I can manage. And how do I get out of my poor financial situation?"

Participant 3
"I got so much better! But I realized that many of my worries were not related to fatigue. The fatigue took so much energy that my other worries seemed to cease to exist. And when I felt better, it was a shock to discover that they were still there even though I was no longer tired. I was extremely tired for so many years, and I still have anxiety about how much the fatigue has stolen from me. Ungrateful perhaps, but that's how it is right now."

Participant 4
"The increase in activity happened too quickly. My body couldn't keep up with the pace, and it resulted in a rather significant crash. Unfortunately, I don't think people with ME can be helped by this type of treatment"

Participant 5
"The negative part was when I crashed due to too much activity."

Participant 6
"I injured myself physically several times during the period when I didn't rest during the day, experienced dizziness and palpitations, had difficulty understanding simple instructions and having normal conversations with my family. I got increased muscle weakness when I didn't rest."

Participant 7
"I've told everyone I meet who sees and feels that the old me is starting to come back. Thanks to you and the doctor who understood that this was 100% the right way to turn around my sleeping. I couldn't have had a better therapist who put up with me."

Participant 8
"My sleep got worse for a while - it goes in periods - but it was probably affected by other factors such as us moving and all the stress that entails."

Questionnaire regarding therapist's feedback on the treatment

When all participants had completed the treatment phase, therapists working in the study were asked to complete a questionnaire regarding their experiences of administrating tCBT. The questionnaire has been translated to English partly using Chat GPT, version 4o and Microsoft Copilot using the prompt "*Translate the following text to English*".

Questionnaire for Therapists After Project Completion

1. To what extent did you find the treatment provided in the study appropriate for the patients you worked with?
2. How did you perceive the treatment platform as part of the intervention?
3. Did you feel that any treatment materials were missing?
4. Was there any part of the treatment that you found difficult to carry out with patients?
5. Did you feel that you received sufficient training and supervision during the course of the treatment?
6. How did you perceive the duration and scope of the treatment period?
7. How did you perceive the treatment format (the 'blended' format)?
8. To what extent do you think it is possible for a patient to achieve the treatment goal (to no longer be hindered in their life by severe fatigue) during the course of the treatment period?
9. Did you receive any feedback from patients during the course of the treatment that you think is important for us to be aware of?
10. Do you have any suggestions for changes that could improve the treatment format and/or content? If so, what are they?

Qualitative analysis of therapist's feedback on the treatment

Methods

Clinician-responses to open-ended questions about the intervention were analyzed using inductive content analysis (Hsieh & Shannon, 2005). One of the authors (DS) coded the data and a second author (FS) checked the coding.

Results

All clinicians (three licensed psychologists and one residential psychologist) completed the questionnaire. Clinicians generally perceived the treatment as appropriate for patients, highlighting its transdiagnostic applicability, though some noted reduced suitability in cases involving greater comorbidity or complex needs. The blended format was appreciated for enhancing continuity, yet clinicians expressed a need for clearer guidance on how to integrate the digital platform effectively.

Table S12. Content analysis of the clinician-responses of the open-ended questions regarding the intervention

Question	Theme	Example quote
1. *To what extent did you find the treatment provided in the study appropriate for the patients you worked with?*	Transdiagnostic suitability confirmed by clinician *n* = 3	I found the treatment to be appropriate for all participants.
	The suitability varies depending on comorbidity and the complexity of the patient's condition. *n* = 1	"Generally suitable for patients with more clear-cut fatigue-related problems. More difficult or less suitable for patients with additional comorbidities."
2. *How did you perceive the treatment platform as part of the intervention?*	The platform simplified the treatment. *n* = 3	"The treatment platform was helpful for administering tasks, forms, and providing a simple means of communication."
	The platform can be improved. *n* = 3	"Some patients complained that the pages froze and that they were not always able to click forward in the treatment on the platform."
3. *Did you feel that any treatment materials were missing?*	The materials were perceived as complete. *n* = 2	"No"
	Additional materials suggested. *n* = 2	"More updates or exercises during the final part of the treatment."
4. *Was there any part of the treatment that you found difficult to carry out with patients?*	Patient characteristics affect outcomes. *n* = 2	"Phase 3 was difficult due to infrequent contact. Neuropsychiatric conditions made implementation more challenging to some extent. It was also more difficult to work with goals when the patient did not have a job to return to."
	Platform could be improved. *n* = 2	"As mentioned, especially toward the end of the treatment (the later phases), it became somewhat more difficult to maintain a good flow with patients in terms of logging in and writing/interacting on the platform.<br>For some, it was also hard to know whether they had actually completed their tasks, as they had not logged in or noted their activity registration.<br>Perhaps there is another way to collect that information that is more user-friendly for patients?"
5. *Did you feel that you received sufficient training and supervision during the course of the treatment?*	Experienced strong clinician support. *n* = 4	"Definitely. I had plenty of time to discuss each patient with other clinicians and with the supervisor."
6. *How did you perceive the duration and scope of the treatment period?*	Appropriate treatment duration. *n* = 3	"Good"
	Individual tailoring of treatment duration. *n* = 1	"I believe the treatment could become even more varied—some patients need a shorter duration, while others may require more than 4–6 months. I also think we need to decide whether the treatment is intended as a kind of 'kick-start' intervention, as is common in primary care, or if the goal is to support patients all the way to full recovery. If it's the latter, I think some patients may need more than 6 months, possibly up to a year, though more spaced out towards the end."
7. *How did you perceive the treatment format (the 'blended' format)?*	The blended format is good. *n* = 3	"I think it adds a lot to be able to both meet the patients in person and maintain contact via the platform during phases where there is a longer interval between sessions."

		More clarity regarding the digital component. $n = 3$	"Good, but personally I found it difficult to figure out how to relate to the online platform. Some patients seemed to engage more online and others more 'in the room,' but I'm not sure on what basis I made that distinction."
8.	*To what extent do you think it is possible for a patient to achieve the treatment goal (to no longer be hindered in their life by severe fatigue) during the course of the treatment period?*	Goal attainment depends on the patient and context. $n = 3$	"A patient without significant comorbidity is, in my view, well positioned to benefit from this."
		Goal attainment is considered fully achievable. $n = 3$	"Very possible!"
9.	*Did you receive any feedback from patients during the course of the treatment that you think is important for us to be aware of?*	Patients request a broader symptom focus. $n = 3$	"Some patients possibly wished for a broader focus on other symptoms as well, particularly pain. I agree, if the aim is to support the patient all the way to recovery. This likely requires further consideration regarding the clinical management of such cases."
10.	*Do you have any suggestions for changes that could improve the treatment format and/or content? If so, what are they?*	Tools to enhance engagement toward the end of the intervention. $n = 3$.	"Add elements that keep the patient more engaged toward the end of the treatment, encouraging them to evaluate their goals and the steps they are taking to reach them. Several patients were afraid of deteriorating, so material addressing this would have been helpful."
		User-friendliness of the digital platform. $n = 1$.	"I think the content is good. Small things I would have liked to change include making the activity form more user-friendly, and allowing users to add other activities, such as running or swimming."

Note: Four clinicians contributed responses. "*n*" refers to times that content were reported. When a single response contained content relevant to multiple categories, it was assigned to each applicable category (e.g.," Worked well. It would have been helpful to have access to all modules in advance in order to review them beforehand.").

Participants feedback on the treatment

Participants were asked to give feedback on the treatment post treatment, the specific question being *"Please provide feedback on the treatment. Write as much as you want about what worked, what didn't work, what could be improved, and what was missing?"*. In total, 13 of 18 participants answered. The answers have been translated to English partly using Chat GPT, version 4o and Microsoft Copilot using the prompt "*Translate the following text to English*".

Participant 1
"*Absolutely fantastic treatment with a mix of tasks such as reading, writing, and reflecting, along with physical meetings where you get feedback on your progress. And now, homework to work on yourself helps you move forward."*

Participant 2
"If you are going to undergo the treatment, it is difficult, like in my case, to work almost full-time and be a single parent with a small child. Some people might need partial sick leave to manage and be able to focus."

Participant 3
"My therapist was very good at encouraging and pushing me. Perhaps I needed him to be a bit more attentive when I felt it was starting to take a toll on my body, but that wasn't really the purpose of the treatment, so maybe that's on me. If I had been in a situation where my body wasn't getting in the way, I would have been very satisfied with the help."

Participant 4
"What made me not follow the treatment during the last part was that I had crashed and became worried that I would get worse if I increased my activity too much, as I can link the crashes in my illness to having had too high activity levels."

Participant 5
"The treatment and support have been good, and I have greatly appreciated the approach. Any shortcomings

that may have existed are on me, as I find it difficult to maintain motivation and dedicate the time required to fully benefit from similar treatments."

Participant 6
"*I would have liked a treatment that took more into account that patients are very different, have varying needs, and were approached based on the fact that fatigue needs to be addressed differently depending on its cause. I am not convinced that stress should be treated in the same way as post-COVID, which stems from infection/virus."*

Participant 7
"It has been important to me that you haven't given up on me, which in turn has meant that I haven't given up on the treatment either. I've had an amazing therapist. I've never felt judged or looked down upon, but rather encouraged to keep going, uplifted, and motivated with tips and tricks. Super satisfied."

Participant 8
"I noticed quite early (actually even before the treatment started) that it wouldn't be helpful for me. I have made similar 'lifestyle changes' when I was in school, which also weren't helpful. But since doctors don't have any better treatments/ideas on how I can get more energetic, I decided to try participating in this study anyway."

Participant 9
"I have received many good tools related to my chronic fatigue. I will take these with me and continue to work on them on my own."

Participant 10
"A very good start. Being encouraged not to sleep during the day resulted in better sleep. The second part, gradually increasing both physical and mental training and social contacts in small steps, made me slowly stronger and stronger and feel progress. This gave me some hope for improvement. The last part, returning to work, was also good. My doctor thought I should work 50% for quite a while due to my background with cancer, etc. This was good. After 6 months, I will increase my working hours a bit more. But I think it was good that I waited with this since my cognitive ability is not yet at its peak. As I said, 6 months was a bit too short for me to fully recover. But considering my age, 61 years, and my tongue pain (after surgery), it might be difficult to fully recover. More doctor contacts would have been good."

Participant 11
"I think the treatment has worked very well. Some of the best things were finally being taken seriously, getting good methods to use in everyday life, and having the opportunity to contact my therapist at any time. As I mentioned earlier, my only wish is that the treatment could have been longer/until I was completely well. It was very good that the treatment could be tailored so much and adapted specifically to my lifestyle."

Participant 12
"Good treatment, nice to have a therapist to discuss with and know what to focus on. However, I felt that the study was a bit geared towards 'couch potatoes' who needed encouragement to get started. I might be off track, but I feel that I need more support to help me slow down and find small breaks in everyday life with three small children, a full-time job, the children's activities, and trying to fit in my own exercise and walks, etc."

Participant 13 (answer has been **shortened** due to its extensive length)

"I think it's important to take each person's current life situation into great consideration. For example, if someone is ill with exhaustion and lives in a relatively stress-free environment, I believe it greatly affects how much the treatment helps. In the case of women, I wish that before the treatment, or at the beginning of the treatment, blood tests were checked...
For me, it has helped to learn to change activities as I easily get stuck and do too much of something. I need to remind myself of this every day. I think the contact with the psychologist has been of great value, it is important to have a clear explanation of what the treatment entails and why it can help. The goals you set during the treatment are great. They create clarity, instill hope, and provide something positive to strive for. I would like some kind of nervous system calming component in the treatment, such as muscle relaxation and calm breathing ...
Being ill for a long time creates all these feelings, and I don't think talk therapy alone helps. A mix of CBT and

perhaps getting the nervous system more relaxed with the help of tactile massage would be beneficial...
It would be great if there were both a physical follow-up after, for example, 3 months after and a digital follow-up where you can both tick off answers and comment. Then, a "handing over" to a health center, psychologist, and/or physiotherapist or other professionals who can take over. When a treatment ends, the feeling of security and care can quickly disappear."

Exploratory effectiveness outcomes

The following measurements were investigated in an exploratory manner to investigate the feasibility of delivering the number of measurements planned to be used in the upcoming RCT.

The following additional exploratory measurements were administrated before (pre, baseline before randomization), during (every third week), and after (post, 6 months from baseline) treatment:

- The Insomnia Severity Index (ISI) 7 items was used to assess insomnia/sleeping problems. The scale range is from 0 to 28, higher scores representing higher insomnia severity (4). In addition to the ISI, one separate item regarding hypersomnia was added, rating how often the responder sleeps more than nine hours during a day, with answers ranging from "Never" to "Always (five times a week or more)".
- The General self-efficacy scale (GSE), 10 items on a 4-pint Likert scale, was used to assess general self-efficacy. The scale range is from 10 to 40, higher scores representing a higher level of self-efficacy (5).

The following additional exploratory measurements were administrated pre and post treatment:

- Checklist Individual Strengths Questionnaire (CIS). The questionnaire consists of 20 items, each rated on a 7-point Likert scale. A total score is calculated by summing the scores of all individual items. The instrument encompasses four subscales: Fatigue Severity (8 items), which assesses the subjective intensity and impact of fatigue; Concentration (5 items), which evaluates difficulties related to sustained attention and cognitive focus; Motivation (4 items), which measures reductions in motivational drive and initiative; and Activity (3 items), which captures the extent of diminished engagement in daily activities (6).
- Patient health questionnaire-9 (PHQ-9) was used to assess depressive symptoms. The questionnaire consists of nine items, each rated on a 4-piont Likert scale. Higher scores representing a higher level of depressive symptoms, 0-4 point as no or minimal depressive symptoms, 5-9 points as mild, 10-14 points as moderate, 15-19 points as moderate/severe, and 20-27 points as severe (7).
- Generalized anxiety disorder 7-item scale (GAD-7) was used to assess level of anxiety. The questionnaire has a maximum score of 21, with higher scores suggesting a higher level of anxiety (8).
- Physical Health Questionnaire-15 (PHQ-15) was used to assess somatic symptoms. The questionnaire has a range between 0-30, with higher scores suggesting more somatic symptoms (9).
- The Perceived Stress Scale (PSS-10) was used to assess perceived stress. The questionnaire measures perceived stress in three areas, life as: unpredictable, uncontrollable, and overloading in the past month. The scale ranges from 0 to 40, with higher scores indicating higher perceived stress (10).
- The Shirom-Melamed Burnout Questionnaire (SMBQ-18) was used to assess burnout. The questionnaire consists of 18 items, each rated on a 7-point Likert scale. The instrument encompasses four subscales: Physical Fatigue, Listlessness, Tension, and Mental Fatigue. The scale ranges from 0 to 7 points, with higher scores indicate more severe symptoms of burnout (11).
- The Self-rated health questionnaire (SRH-5) was used to assess self-rated health. The questionnaire contains one item, five responses, rating the responder's perception of their general health from "Poor" to "Excellent". The scale ranges from 0-4 with higher scores indicating better perceived general health (12).
- The World Health Organization Disability Assessment Schedule 2.0 (WHODAS 2.0), 12-item version, was used to assess functional disability. Each item is rated on a 5-point Likert scale, and results are presented in total points (scale range 0-60) and domain points (0-100), with higher scores indicating higher disability (13).

Descriptive statistics of additional clinical outcomes

Table S13 displays reported values (mean, SD) for all additional outcomes at pre and post, including p-value and effect sizes. Effectiveness outcomes were analyzed using intention-to-treat linear mixed regression models, with significance level set at 0·05. Effect sizes were calculated as repeated measures Cohen's d using the mean pre–post difference divided by the standard deviation of the difference scores. Effect sizes, Cohen's d, has been adjusted so that positive sign indicates improvement.

Table S13. Observed means, pre-post effect size, and linear mixed model estimates of secondary outcome measures.

Scale / Subscale	Pre	Post	Slope/b [95% CI]	*p*	Cohen's d [95% CI]
CIS - Physical Activity	17·56 (4·00)	12·67 (5·12)	0·20 [0·30, 0·11]	<0·001	1·00 [0·46, 1·54]
CIS - Concentration	27·67 (5·58)	20·28 (6·53)	0·31 [0·43, 0·18]	<0·001	1·15 [0·57, 1·74]
CIS - Motivation	17·61 (6·22)	11·83 (5·87)	0·24 [0·33, 0·16]	<0·001	0·91 [0·54, 1·28]
CIS - Total Score	115·39 (13·97)	81·89 (24·55)	1·40 [1·86, 0·93]	<0·001	1·54 [0·80, 2·27]
GAD-7	4·00 (3·09)	3·72 (3·69)	0·01 [0·05, -0·03]	0·572	-0·38 [-0·70, -0·06]
GSE Self Efficacy	27.17 (5·58)	29·72 (6·78)	0·10 [0·05, 0·16]	<0·001	0·38 [-0·70, -0·06]
Hypersomnia	3.72 (1·27)	2·50 (1·58)	0·05 [0·09, 0·01]	0·013	0·81 [0·07, 1·56]
ISI	12.94 (4·72)	7·83 (5·00)	0·20 [0·26, 0·13]	<0·001	1·00 [0·51, 1·50]
PHQ-15	13.06 (5·34)	10·67 (5·34)	0·10 [0·17, 0·03]	0·011	0·43 [0·11, 0·75]
PHQ-9	13·72 (3·88)	6·50 (3·59)	0·30 [0·38, 0·22]	<0·001	1·84 [1·07, 2·62]
PSS-10	21·50 (4·58)	15·72 (7·03)	0·24 [0·35, 0·13]	<0·001	0·88 [0·41, 1·34]
SMBQ - Listlessness	5·92 (0·89)	4·40 (1·56)	0·06 [0·09, 0·03]	<0·001	1·11 [0·44, 1·78]
SMBQ - Physical Fatigue	5·71 (0·92)	4·19 (1·57)	0·06 [0·09, 0·03]	<0·001	1·09 [0·45, 1·72]
SMBQ - Mental Fatigue	5·53 (1·08)	3·76 (1·50)	0·07 [0·10, 0·05]	<0·001	1·27 [0·62, 1·91]
SMBQ - 18 Total Score	5·69 (0·81)	4·10 (1·45)	0·07 [0·09, 0·04]	<0·001	1·26 [0·56, 1·96]
SRH-5	1·28 (0·96)	2·17 (0·92)	0·04 [0·01, 0·06]	0·006	0·90 [1·60, 0·21]
WHODAS 2.0 (0-100)	49·77 (14·06)	30·09 (18·73)	0·82 [1·15, 0·49]	<0·001	1·11 [0·55, 1·67]

Note: CIS, Checklist Individual Strength. GAD-7, Generalized anxiety disorder 7-item scale. GSE Self Efficacy, The General self-efficacy scale. ISI, The Insomnia Severity Index. PHQ-15, Physical Health Questionnaire-15. PHQ-9, Patient health questionnaire-9. PSS-10, The Perceived Stress Scale. SMBQ, The Shirom-Melamed Burnout Questionnaire. SRH-5, The Self-rated health questionnaire. WHODAS 2.0, The World Health Organization Disability Assessment Schedule 2.0.

Process measures (every third week)

Table S14 displays mean scores of CIS-F, GSE, ISI and CBRQ at all measured timepoints in the study. At timepoint "Week 15" 17 out of 18 (94%) participants completed the process measurement. At timepoints "Week 15" and "Week 21" 16 out of 18 (89%) participants completed the process measurement 89%. There was no data attrition at the other timepoints.

Table S14. Reported values (mean, SD) over all timepoints in the study.

Scale / Subscale	*Pre*	*Week 3*	*Week 6*	*Week 9*	*Week 12*	*Week15*	*Week 18*	*Week 21*	*Post*
CIS - Fatigue	50·44 (5·49)	45·89 (7·93)	43·17 (9·53)	41·28 (8·32)	40·72 (9·88)	38·35 (9·28)	43·00 (8·97)	39·69 (9·93)	37·11 (11·80)
GSE Self Efficacy	27·17 (5·58)	27·78 (5·39)	28·00 (6·13)	29·83 (5·22)	29·61 (5·83)	28·29 (5·36)	30·38 (3·36)	30·75 (3·66)	29·72 (6·78)
ISI Insomnia	12·94 (4·72)	12·72 (5·22)	11·17 (5·82)	8·72 (6·08)	8·94 (5·53)	8·06 (3·73)	9·19 (4·10)	8·69 (4·60)	7·83 (5·00)
CBRQ - All or Nothing	8·56 (2·18)	7·83 (2·33)	7·11 (1·84)	6·56 (2·04)	5·78 (2·41)	6·06 (1·52)	6·31 (1·58)	6·19 (1·72)	6·00 (2·30)
CBRQ - Damage Beliefs	7·39 (1·61)	5·50 (1·54)	5·06 (1·76)	4·94 (1·83)	4·89 (1·64)	4·88 (2·18)	4·56 (2·42)	4·62 (2·53)	4·39 (2·99)
CBRQ - Embarrassment Avoidance	4·28 (2·78)	5·06 (2·92)	3·89 (3·18)	4·17 (3·29)	3·83 (3·75)	3·12 (3·31)	3·38 (2·83)	3·25 (2·59)	2·39 (3·22)
CBRQ - Fear Avoidance	6·50 (3·19)	6·28 (2·42)	5·44 (2·43)	5·44 (2·97)	5·33 (3·05)	5·00 (3·14)	5·44 (2·73)	4·62 (3·16)	4·89 (3·77)
CBRQ - Resting Behavior	7·89 (2·32)	4·22 (2·51)	3·22 (2·02)	2·89 (2·52)	2·61 (2·85)	2·65 (2·80)	2·50 (2·53)	2·88 (3·05)	2·33 (3·09)
CBRQ - Symptom Focusing	8·00 (2·30)	7·56 (1·65)	6·39 (2·09)	5·44 (1·65)	5·11 (2·08)	5·00 (2·65)	5·19 (1·91)	4·62 (2·42)	4·67 (2·81)

Note: CIS, Checklist Individual Strength. GSE, Self Efficacy. ISI, The Insomnia Severity Index. CBRQ, Cognitive and Behavioral Responses to Symptoms Questionnaire.

Transdiagnostic cognitive behavioral therapy for severe and persistent fatigue–a feasibility study in primary care

Graphs CIS-F and CBRQ: Process measures (every third week)

Figure S2: **CIS-F and CBRQ over all timepoints in the study.**

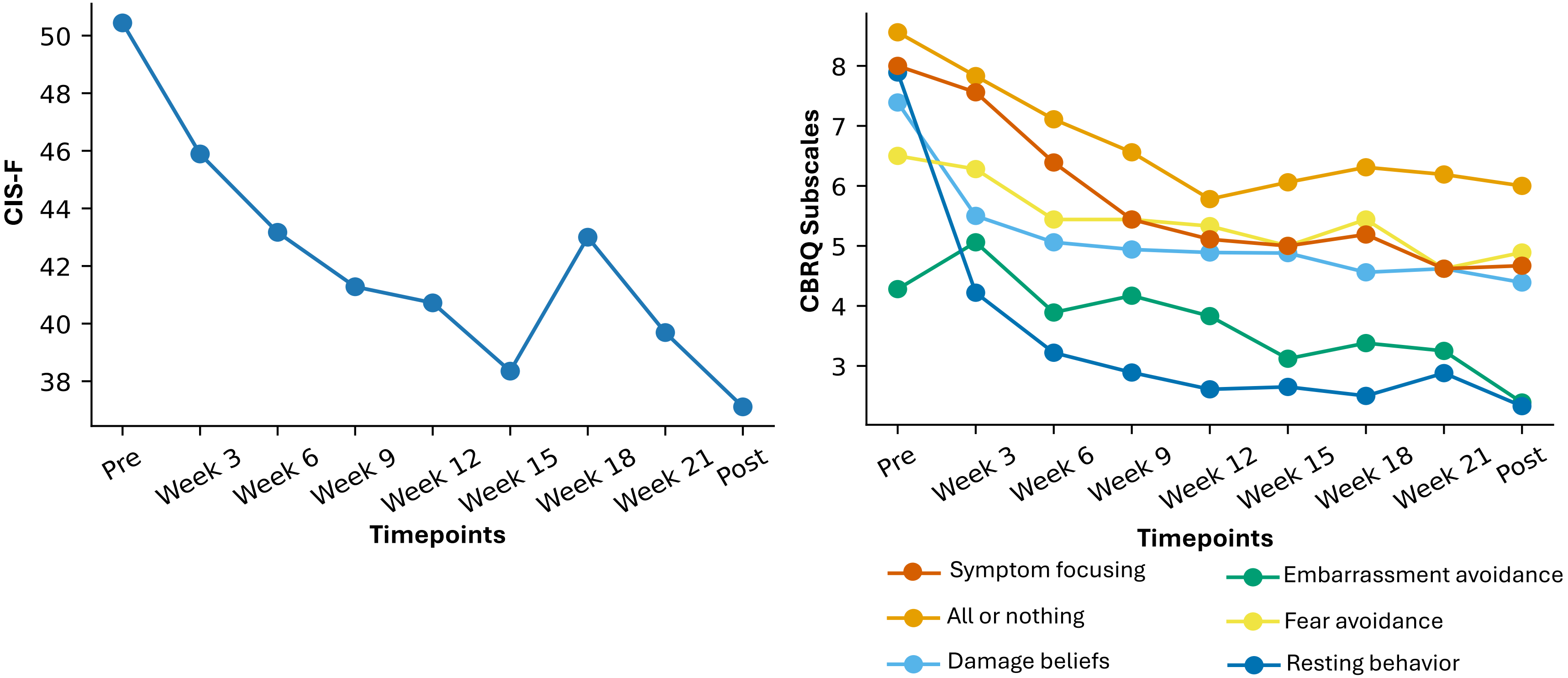

Figure S2 displays mean scores of CIS-F and CBRQ at all measured timepoints in the study. At timepoint “Week 15” 17 out of 18 (94%) participants completed the process measurement. At timepoints “Week 15” and “Week 21” 16 out of 18 (89%) participants completed the process measurement 89%. There was no data attrition at the other timepoints. CIS, Checklist Individual Strength. CBRQ, Cognitive and Behavioral Responses to Symptoms Questionnaire.

Transdiagnostic cognitive behavioral therapy for severe and persistent fatigue–a feasibility study in primary care

CONSORT Checklist for pilot or feasibility trial

CONSORT 2010 checklist of information to include when reporting a pilot or feasibility trial*

Section/Topic	Item No	Checklist item	Reported on page No
**Title and abstract**			
	1a	Identification as a pilot or feasibility randomised trial in the title	1
	1b	Structured summary of pilot trial design, methods, results, and conclusions (for specific guidance see CONSORT abstract extension for pilot trials)	1
**Introduction**			
Background and objectives	2a	Scientific background and explanation of rationale for future definitive trial, and reasons for randomised pilot trial	3-4
	2b	Specific objectives or research questions for pilot trial	4
**Methods**			
Trial design	3a	Description of pilot trial design (such as parallel, factorial) including allocation ratio	4
	3b	Important changes to methods after pilot trial commencement (such as eligibility criteria), with reasons	18
Participants	4a	Eligibility criteria for participants	6
	4b	Settings and locations where the data were collected	4-5
	4c	How participants were identified and consented	4-5
Interventions	5	The interventions for each group with sufficient details to allow replication, including how and when they were actually administered	6-7
Outcomes	6a	Completely defined prespecified assessments or measurements to address each pilot trial objective specified in 2b, including how and when they were assessed	8-10
	6b	Any changes to pilot trial assessments or measurements after the pilot trial commenced, with reasons	N/A

	6c	If applicable, prespecified criteria used to judge whether, or how, to proceed with future definitive trial	N/A
Sample size	7a	Rationale for numbers in the pilot trial	4-5
	7b	When applicable, explanation of any interim analyses and stopping guidelines	N/A
Randomisation:			
Sequence generation	8a	Method used to generate the random allocation sequence	N/A
	8b	Type of randomisation(s); details of any restriction (such as blocking and block size)	N/A
Allocation concealment mechanism	9	Mechanism used to implement the random allocation sequence (such as sequentially numbered containers), describing any steps taken to conceal the sequence until interventions were assigned	N/A
Implementation	10	Who generated the random allocation sequence, who enrolled participants, and who assigned participants to interventions	N/A
Blinding	11a	If done, who was blinded after assignment to interventions (for example, participants, care providers, those assessing outcomes) and how	N/A
	11b	If relevant, description of the similarity of interventions	N/A
Statistical methods	12	Methods used to address each pilot trial objective whether qualitative or quantitative	10
**Results**			
Participant flow (a diagram is strongly recommended)	13a	For each group, the numbers of participants who were approached and/or assessed for eligibility, randomly assigned, received intended treatment, and were assessed for each objective	10-11
	13b	For each group, losses and exclusions after randomisation, together with reasons	11
Recruitment	14a	Dates defining the periods of recruitment and follow-up	10-11
	14b	Why the pilot trial ended or was stopped	N/A
Baseline data	15	A table showing baseline demographic and clinical characteristics for each group	11-12
Numbers analysed	16	For each objective, number of participants (denominator) included in each analysis. If relevant, these numbers	13

		should be by randomised group	
Outcomes and estimation	17	For each objective, results including expressions of uncertainty (such as 95% confidence interval) for any estimates. If relevant, these results should be by randomised group	14
Ancillary analyses	18	Results of any other analyses performed that could be used to inform the future definitive trial	Supplementary material
Harms	19	All important harms or unintended effects in each group (for specific guidance see CONSORT for harms)	13
	19a	If relevant, other important unintended consequences	N/A
**Discussion**			
Limitations	20	Pilot trial limitations, addressing sources of potential bias and remaining uncertainty about feasibility	17-18
Generalisability	21	Generalisability (applicability) of pilot trial methods and findings to future definitive trial and other studies	18
Interpretation	22	Interpretation consistent with pilot trial objectives and findings, balancing potential benefits and harms, and considering other relevant evidence	18-19
	22a	Implications for progression from pilot to future definitive trial, including any proposed amendments	17-18
**Other information**			
Registration	23	Registration number for pilot trial and name of trial registry	2/4
Protocol	24	Where the pilot trial protocol can be accessed, if available	2/4
Funding	25	Sources of funding and other support (such as supply of drugs), role of funders	19
	26	Ethical approval or approval by research review committee, confirmed with reference number	4

Citation: Eldridge SM, Chan CL, Campbell MJ, Bond CM, Hopewell S, Thabane L, et al. CONSORT 2010 statement: extension to randomised pilot and feasibility trials. BMJ. 2016;355. This is an Open Access article distributed in accordance with the terms of the Creative Commons Attribution (CC BY 3.0) license (http://creativecommons.org/licenses/by/3.0/), which permits others to distribute, remix, adapt and build upon this work, for commercial use, provided the original work is properly cited.

*We strongly recommend reading this statement in conjunction with the CONSORT 2010, extension to randomised pilot and feasibility trials, Explanation and Elaboration for important clarifications on all the items. If relevant, we also recommend reading CONSORT extensions for cluster randomised trials, non-inferiority and equivalence trials, non-pharmacological treatments, herbal interventions, and pragmatic trials. Additional extensions are forthcoming: for those and for up-to-date references relevant to this checklist, see www.consort-statement.org.

Transdiagnostic cognitive behavioral therapy for severe and persistent fatigue–a feasibility study in primary care

References

1. Barber JP, Liese, B.S., and Abrams MJ. Development of the Cognitive Therapy Adherence and Competence Scale. Psychother Res. 01 juni 2003;13(2):205–21.

2. Harned MS, Schmidt SC, Korslund KE, Gallop RJ. Development and Evaluation of a Pragmatic Measure of Adherence to Dialectical Behavior Therapy: The DBT Adherence Checklist for Individual Therapy. Adm Policy Ment Health Ment Health Serv Res. 01 september 2023;50(5):734–49.

3. Okkersen K, Jimenez-Moreno C, Wenninger S, Daidj F, Glennon J, Cumming S, m.fl. Cognitive behavioural therapy with optional graded exercise therapy in patients with severe fatigue with myotonic dystrophy type 1: a multicentre, single-blind, randomised trial. Lancet Neurol. 01 augusti 2018;17(8):671–80.

4. Oswald I. Insomnia — Psychological Assessment and Management By Charles M. Morin New York: Guilford Press. 1993. 238 pp. US $24.25. Br J Psychiatry. juni 1995;166(6):830–830.

5. Chen G, Gully SM, Eden D. Validation of a New General Self-Efficacy Scale. Organ Res Methods. 01 januari 2001;4(1):62–83.

6. Worm-Smeitink M, Gielissen M, Bloot L, van Laarhoven HWM, van Engelen BGM, van Riel P, m.fl. The assessment of fatigue: Psychometric qualities and norms for the Checklist individual strength. J Psychosom Res. 01 juli 2017;98:40–6.

7. Kroenke K, Spitzer RL, Williams JBW. The PHQ-9. J Gen Intern Med. 01 september 2001;16(9):606–13.

8. Spitzer RL, Kroenke K, Williams JBW, Löwe B. A Brief Measure for Assessing Generalized Anxiety Disorder: The GAD-7. Arch Intern Med. 22 maj 2006;166(10):1092–7.

9. Interian A, Allen LA, Gara MA, Escobar JI, Díaz-Martínez AM. Somatic Complaints in Primary Care: Further Examining the Validity of the Patient Health Questionnaire (PHQ-15). Psychosomatics. 01 september 2006;47(5):392–8.

10. Nordin M, Nordin S. Psychometric evaluation and normative data of the Swedish version of the 10-item perceived stress scale. Scand J Psychol. 2013;54(6):502–7.

11. Almén N, Jansson B. The reliability and factorial validity of different versions of the Shirom-Melamed Burnout Measure/Questionnaire and normative data for a general Swedish sample. Int J Stress Manag. november 2021;28(4):314–25.

12. Eriksson I, Undén AL, Elofsson S. Self-rated health. Comparisons between three different measures. Results from a population study. Int J Epidemiol. 01 april 2001;30(2):326–33.

13. Axelsson E, Lindsater E, Ljotsson B, Andersson E, Hedman-Lagerlof E. The 12-item Self-Report World Health Organization Disability Assessment Schedule (WHODAS) 2.0 Administered Via the Internet to Individuals With Anxiety and Stress Disorders: A Psychometric Investigation Based on Data From Two Clinical Trials. JMIR Ment Health. 2017;4(4):e58.
